# Supplementary material for: Psychometric evaluation of the Chinese version of the Person-centred Climate Questionnaire - Staff version (PCQ-S)
Source: BMJ Open. 2017 Aug 28;7(8):e017250. doi: 10.1136/bmjopen-2017-017250 (PMC5724089; doi:10.1136/bmjopen-2017-017250)
Supplement: Author's manuscript [file bmjopen-2017-017250.draft_revisions.pdf]

BMJ Open is committed to open peer review. As part of this commitment we make the peer review history of every article we publish publicly available.

When an article is published we post the peer reviewers' comments and the authors' responses online. We also post the versions of the paper that were used during peer review. These are the versions that the peer review comments apply to.

The versions of the paper that follow are the versions that were submitted during the peer review process. They are not the versions of record or the final published versions. They should not be cited or distributed as the published version of this manuscript.

BMJ Open is an open access journal and the full, final, typeset and author-corrected version of record of the manuscript is available on our site with no access controls, subscription charges or pay-per-view fees (http://bmjopen.bmj.com).

If you have any questions on BMJ Open's open peer review process please email info.bmjopen@bmj.com

BMJ Open

Psychometric evaluation of the Chinese version Person-centred Climate Questionnaire - staff

Journal:	*BMJ Open*
Manuscript ID	bmjopen-2017-017250
Article Type:	Research
Date Submitted by the Author:	12-Apr-2017
Complete List of Authors:	Cai, Le; School of Public Health<br>Ahlström, Gerd; Lunds Universitet<br>Tang, Pingfen; Kunming Medical University<br>Ma, Ke; The third people's hospital of Kunming<br>Edvardsson, David; La Trobe University School of Nursing and Midwifery<br>Behm, Lina; Lunds Universitet<br>Fu, Haiyan; the third people's hospital of Kunming<br>Zhang, Jie; the third people's hospital of Kunming<br>Yang, Jiqun; the third people's hospital of Kunming
**Primary Subject Heading**:	Nursing
Secondary Subject Heading:	Geriatric medicine, Nursing
Keywords:	GERIATRIC MEDICINE, Quality in health care < HEALTH SERVICES ADMINISTRATION & MANAGEMENT, PALLIATIVE CARE

SCHOLARONE™
Manuscripts

For peer review only

Psychometric evaluation of the Chinese version Person-centred Climate Questionnaire - staff

Cai Le [1], Gerd Ahlström [2], Tang Pingfen [1], Ma Ke [3], David Edvardsson [4], Lina Behm [2], Fu Haiyan [3], Zhang Jie [3] and Yang Jiqun [3]

Address: [1] 1168 Yu Hua Street Chun Rong Road, Cheng Gong New City, School of Public Health, Kunming Medical University, Kunming 650500, China;

[2] Department of Health Sciences, Faculty of Medicine, Lund University, Sweden;

[3] Division of palliative care, the third people's hospital of Kunming, China;

[4]Austin Health/Northern Health Clinical Schools of Nursing College of Science, Health and Engineering, La Trobe University, Australia

Email address:

Cai Le * : 1018606825@qq.com;

Gerd Ahlström: gerd.ahlstrom@med.lu.se;

Tang Pingfen: yangyi57@vip.126.com;

Ma Ke: kmmake@126.com;

David Edvardsson: D.Edvardsson@latrobe.edu.au;

Lina Behm: Lina.behm@med.lu.se;

Fu Haiyan: fuhaiyan0622@163.com;

Zhang Jie: 13888437137@126.com;

Yang Jiqun: 1181780508@qq.com

* Corresponding author

For peer review only

Abstract

Objectives

The aim of the study was to conduct a cross-cultural adaptation of the English version Person-centred Climate Questionnaire – staff version (PCQ-S) for Chinese health care staff and to evaluate the psychometric properties of the translated Chinese version in a hospital context.

Design

This was a cross-sectional design. The 14-item English PCQ-S was translated and back translated using established procedures. Construct validity and reliability including internal consistency and test-retest reliability were assessed among hospital staffs. Construct validity was tested using Principal component analysis (PCA). Internal consistency was tested using Cronbach's alpha. Test-retest reliability was evaluated through the Pearson correlation coefficient (r) and intra-class correlation coefficient (ICC).

Setting

This study was conducted in three hospitals in Kunming, the capital of the Yunnan province in south-west China.

Participants

A sample of hospital staff (n=163) on duty at the departments of palliative care in three hospitals of Kunming consented to participate in the study.

Results

The results confirmed that the 14-item Chinese PCQ-S consisted of the three subscales shown in other language versions. It showed strong internal consistency through a Cronbach's alpha of 0.94 for the total scale, 0.87 for the safety subscale, 0.90 for the everydayness subscale and 0.88 for the community subscale. The Chinese PCQ-S had high test-retest reliability as evidenced by a high correlation coefficient on all scale levels between test and retest scores, on 'a climate of safety' (r = 0.88, P<0.01), 'a climate of everydayness' (r=0.91, P<0.01), 'a climate of community' (r=0.79, P<0.01) and on overall scale scores (r=0.93, P<0.01). The ICC to evaluate

For peer review only - http://bmjopen.bmj.com/site/about/guidelines.xhtml

the test-retest reliability was 0.93 (95% CI: 0.91- 0.95).

Conclusions

The Chinese version of PCQ-S is a seemingly valid and reliable tool, which showed satisfactory reliability and validity for assessing staff perceptions of the person-centredness climate in Chinese hospital environments.

Keywords: Person-centred care; hospitals; nursing; questionnaire; scale development; China

Article summary

Strengths and limitations of this study

- The first study to validate the PCQ-S in an Asian population.
- High response rate (90%) in this study.
- Convenience sampling method may limit the ability to generalize the results.
- The Chinese PCQ-S has been tested only in this hospital environment.

For peer review only - http://bmjopen.bmj.com/site/about/guidelines.xhtml

Introduction

Population aging is a global phenomenon and has become a significant public health problem worldwide. Along with the high-speed economic growth and demographic change in the last two decades, China is becoming one of the most rapidly ageing countries in the world. The proportion of older people aged 60 years or more was 13.3 % in 2010 [1], and is projected to reach 32.8 % by 2050 [2]. Studies have indicated that older people are more likely to suffer from various diseases, and more particularly chronic disease and comorbidity, which is more difficult to cure due to complexity and coexistence [3]. Chronic diseases may result in disability in older people; therefore, the rising number of older people increases the demand for hospitalization and special care and supports from multiple care professionals and providers [4]. This presenting a key challenge for Chinese healthcare systems to provide high quality care for this group.

In recent decades, person-centred care (PCC) has become recognized as a quality focus of elderly care services, which emphasizes the individual's perspective and active participation in the care process [5]. PCC as a concept implies in various ways assisting an individual to be able to be a "whole" human being, by encouraging them to participate in decisions and adjusting the physical environment and the content of the care to fit the needs of each individual. It is defined as 'valuing people as individuals' in delivering health care [6] and are based on people's subjective experience of illness instead of the disease [7-9]. The care process becomes the foundation for how PCC should be provided and the relationship between the professional caregiver and the care recipient becomes essential [10-12]. Person-centredness is now regarded as a central feature of high quality long-term care for older persons. As such PCC must be a part of the care organisation priorities, and the system need to support and sustain this change through policy and procedures, job descriptions, and education [13]. PCC improves autonomy in elderly care through its focus on individual care plans and support for next of kin who are seen as important resources [14]. There is evidence to indicate that the person-centredness of a setting is

For peer review only - http://bmjopen.bmj.com/site/about/guidelines.xhtml

associated with staff's satisfaction with work [15], and staff perceptions of and relationships with patients are crucially important to quality care. Also, for the older person, a person-centered setting have shown to increased wellbeing and decreased discomfort [16, 17].

Edvardsson and colleagues have developed a Swedish language Person-centred Climate Questionnaire – staff version (PCQ-S) for evaluating to what extent the climate of care environments are experienced as being person-centred by staff [18]. This instrument has been translated from Swedish into Norwegian [19] and English [20]. The original, as well as the translated Norwegian and English scales have been demonstrated to be valid and reliable tools for assessing staff perceptions of person-centredness. However, there exists no Chinese version of the PCQ-S so far, which presents a barrier for measuring and developing of person-centered care and for conducting further studies in China with possibilities to make international comparisons. Therefore, the purpose of this study was to conduct a cross-cultural adaptation of the English version of PCQ-S for Chinese health care staff and to evaluate the psychometric properties of the translated Chinese version in a hospital context.

Methods

Instrument

The English PCQ-S questionnaire consists of 14 items and has three subscales (a climate of safety, everydayness, and community) [19]. A climate of safety is measured through items 1-5, everydayness is measured through items 6-10, and community is measured through items 11-14. Scoring is performed on a 6-point Likert-type scale, ranging from 0 (No, I disagree completely) to 5 (Yes, I agree completely). Aggregated scores are calculated using simple sum scores at subscale and total scale levels, which range from 0 to 70, with higher scores indicating a setting perceived as being more person-centred. The English PCQ-S has previously been used and tested in hospital settings, and demonstrated to be a valid and reliable

For peer review only - http://bmjopen.bmj.com/site/about/guidelines.xhtml

tool for assessing staff perceptions of the unit person-centredness [20].

Translation and cross-cultural adaptation of the PCQ-S

The translation and cross-cultural adaptation was carried out according to previously published international test commission guidelines [21, 22]. First, forward translation from English to Chinese was performed independently by three native Chinese, two of them were university graduates with Public Health background and the last person was a physician familiar with palliative care. A consensus version was obtained after a discussion between the three translators. Second, the consensus version was back translated into English by two bilingual translators blinded to the procedures of the forward translation. Finally, a thorough comparison of the original, translated and back-translated versions was conducted by an expert committee, which consisted of all translators, three palliative care physicians and two university professors. Discrepancies in translations was discussed and resolved, a few wordings were adapted to the Chinese cultural setting, and a consensus pre-final version was reached. A final Chinese version was generated after pre-testing through face-validity the pre-final version on 10 staff from a municipal hospital of Kunming, no any changes was made after the pre-testing.

Sample and participants

Three municipal hospitals of Kunming, the capital of Yunnan province in south-west China, were selected through a convenience sampling method. Their participation was granted by the hospital directors. All staff (n=182) on duty at the departments of palliative care in these three hospitals were considered eligible for participation, and invited to complete the Chinese PCQ-S questionnaire. The eligible staff received oral information about the study and 163 consented to participate, representing an overall response rate of 90%. The participants completed questionnaires for both the test and retest assessments.

Data collection

For peer review only

For peer review only - http://bmjopen.bmj.com/site/about/guidelines.xhtml

Demographic data was collected along with the questionnaire survey including staff age, gender, level of education and duration of work experience. Two university graduates distributed questionnaires to all staff, and completed questionnaires were anonymously collected on site. To examine the test-retest reliability, all participants were asked to complete the same PCQ-S questionnaire 1 week later. Data were collected during October and November in 2016.

Psychometric evaluation

No variable had missing values. All complete data were included in the analysis. Construct validity was estimated using principal component analysis (PCA) with varimax orthogonal rotation. Bartlett's test of sphericity was used to assess whether the correlation between items was adequate based on a criterion of $p< 0.0001$. The Kaiser-Meyer-Olkin (KMO) statistic was used to measure sample adequacy based on a criterion of ≥0.7. Principal components were extracted when Kaiser's criterion of eigenvalues was ≥1. A component loading cut off of 0.5 was used to decide if an item loaded on a specific component. PCA with varimax orthogonal rotation was performed to ensure independence of the items.

Reliability testing included assessments of internal consistency and test-retest reliability. Internal consistency for total and subscale scores was estimated using the Cronbach's alpha coefficient, and the cut-off scores for acceptable reliability was set to item-total correlations of ≥0.5 and that the Cronbach's alpha would not be increased by item deletion [23]. Test–retest reliability was evaluated through the Pearson correlation coefficient (r) and a single measure two-way mixed effects model intra-class correlation (ICC), where an ICC >0.80 was taken to indicate satisfactory reliability [24]. The paired t-test was used to determine whether mean scores of the test and retest questionnaires differed significantly. All statistical significance decisions were based on two-tailed P values of <0.05. All data analyses were conducted using the SPSS 17.0 software.

For peer review only - http://bmjopen.bmj.com/site/about/guidelines.xhtml

Results

Demographic characteristics of the study group

Table 1 shows the demographic characteristics of the study group. The sample consisted of 92.6% female and 7.4% male staff. The mean age was 31.6 years (SD ± 10.1) with an average length of work experience in healthcare of 8.1 years (SD ± 7.4). The percentage of ethnic minorities was more than one fourth. Most participants were registered nurses (67.2%) and enrolled nurses (17.8%). About one third (33.2%) of the participants had a bachelor's degree or higher (see Table 1).

Construct validity

The results of the PCA with Bartlett's test ($p< 0.0001$) and the KMO Measure (0.91) indicated that correlations between items were sufficiently large to perform the PCA. As shown in Table 2, the PCA resulted in a three-component rotated solution that explained 73.3% of the total variance in data. The first and second component consisted of five items (loadings between 0.58 and 0.83 vs. loadings between 0.68 and 0.82), where the first component confirmed the sub-scale 'A climate of safety' and where the second component confirmed the sub-scale 'A climate of everydayness' in the setting. The third component comprised four items (loadings between 0.64 and 0.87), and confirmed the sub-scale 'A climate of community'.

Reliability

Table 2 shows that the Cronbach's alpha coefficient of the 14-item Chinese PCQ-S was 0.89 for the total scale, 0.87 for the safety subscale, 0.90 for the everydayness subscale and 0.88 for the community subscale, indicating a strong internal consistency reliability overall. Furthermore, the corrected item-total correlations for all items ranged from 0.54 to 0.79, indicating that each item correlated adequately with the total score and thus that the scale is homogenous without any item being redundant (Table 3).

Table 4 presents the results from the test-retest reliability assessment of the Chinese

For peer review only - http://bmjopen.bmj.com/site/about/guidelines.xhtml
For peer review only

PCQ-S. According to the Pearson's correlation coefficient analyses, the Chinese PCQ-S indicated high correlation between test and retest on all scale levels, for with the sub-scale 'a climate of safety' ($r = 0.88$, $P<0.01$), 'a climate of everydayness' ($r=0.91$, $P<0.01$), and 'a climate of community' ($r=0.79$, $P<0.01$) as well as on the overall scale scores between test and retest ($r=0.93$, $P<0.01$). A paired t-test also confirmed that there was no significant difference between the mean scores of the PCQ-S at the test and retest ratings ($P>0.05$). The ICC of the total score between the test and retest times was 0.97, providing further support that the scale had satisfactory test–retest reliability.

Discussion

The original PCQ-S created by Edvardsson and colleagues [18, 19] is one of the most commonly used instruments to evaluate to what extent the climate of care environments are experienced as being person-centred by staff. It has been validated with older persons being cared for in hospitals, and shown to have satisfactory psychometric properties. To date, three different language versions of the PCQ-S have been validated in Western countries, and shown to have appropriate psychometric properties in Australian and Norwegian populations [20, 25]. However, this is the first study to validate the PCQ-S in an Asian population, and the results of the present study indicated that the cross-culturally adapted Chinese version PCQ-S showed excellent reliability and validity for evaluating staff perceptions of person-centredness in Chinese hospital contexts, which enables further studies and international comparisons.

In this study, the English PCQ-S was cross-culturally adapted into the Chinese version and showed satisfactory psychometric properties (construct validity, test-retest reliability and internal consistency). Construct validity was estimated using PCA with varimax orthogonal rotation resulted in a stable three-factor solution explaining 73.3% of the total variance. The ICC for the overall Chinese PCQ-S scale was 0.97 and for the three subscales was 0.93, 0.95 and 0.92, demonstrating the test-retest

For peer review only - http://bmjopen.bmj.com/site/about/guidelines.xhtml

reliability of the overall scale and different domains were all found to be excellent. Furthermore, strong internal consistency of the Chinese PCQ-S was demonstrated as evidenced through a Cronbach's alpha of 0.89 for the total scale, 0.87 for the safety subscale, 0.90 for the everydayness subscale and 0.88 for the community subscale.

This Chinese version PCQ-S showed the same sub-scale structure as the Swedish and Norwegian versions - a structure with the three subscales described above (a climate of safety, everydayness and community) consisted of 14 items. Even though the original English version PCQ-S described a slightly different structure consisting of three subscales, the instrument developers have recently suggested to keep with the three subscale structure for scoring and comparison purposes as this confirms the theory on which the scale rests [18].

In the Chinese PCQ-S, the ICC (0.97) and Cronbach's alpha for the total scale (0.94) was much higher than recorded in Swedish (0.51 vs. 0.88) and English (0.75 vs. 0.89) version, and the Cronbach's alpha for the total scale was also higher than the Norwegian version (0.92), indicating the Chinese PCQ-S had stronger test-retest reliability and internal consistency compared to other language versions of the PCQ-S. The results demonstrated that the Chinese PCQ-S has good reproducibility and well maintain the properties of the original version which can be used in Chinese hospital environments.

The following limitations of the present study should be noted. First, the study employed a convenience sampling method to select staff in palliative care from public hospitals, which may limit the ability to generalize the results to a larger population of staff in Chinese hospitals and beyond. Second, the Chinese PCQ-S questionnaire has been tested only in this hospital environment, further psychometric testing of the scale in other settings like nursing homes would be helpful to enable further rigorous comparisons of Chinese PCQ-S in different contexts and settings.

For peer review only - http://bmjopen.bmj.com/site/about/guidelines.xhtml

Conclusion

The 14-item Chinese version PCQ-S is a cross-culturally adapted version of the English PCQ-S, which showed excellent psychometric properties in terms of reliability and validity for evaluating staff perceptions of the person-centredness in Chinese hospital environments.

Ethical approval and consent to participate

This study was approved by the Ethics Committee of Kunming Medical University. Oral informed consent was obtained from all persons participating in the study.

Availability of data and material

The datasets used and/or analysed during the current study is available from the corresponding author on reasonable request.

Competing interests

The authors declare that there are no conflicts of interest.

Funding

The study was supported by grants from the National Natural Science Fund of China (Grant number: 81611130077), the Swedish Research Council (Grant number: 2015-06243) and Major Union Specific Project Foundation of Yunnan Provincial Science and Technology Department and Kunming Medical University (2016).

Authors' contributions

CL (Kunming Medical University, China) was responsible for the study design, data analysis, and drafting the paper. GA (Lund University, Sweden), TP (Kunming Medical University, China) and MK (the third people's hospital of Kunming, China) contributed to the study design and provided comments on the paper during the writing process. DE (La Trobe University, Australia) and LB (Lund University, Sweden) provided comments on the paper during the writing process. FH, ZJ and JY

For peer review only - http://bmjopen.bmj.com/site/about/guidelines.xhtml

(the third people's hospital of Kunming, China) were responsible for the data collection.

Acknowledgements

We would like to thank Ms Yang Jingjing and Ms Wu Chao, two postgraduates of School of Public Health, Kunming Medical University, who helped us with data collection and Magnus Persson, Lund University, Sweden for providing comments on the paper.

For peer review only

For peer review only - http://bmjopen.bmj.com/site/about/guidelines.xhtml

References

1. **Sixth National Population Census of the People's Republic of China**. Beijing, China: National Bureau of Statistics of China; 2011.
2. **World population prospects: the 2012 revision.** New York, United States: Department of Economic and Social Affairs (DoEaSA), Population Division, United Nations; 2013.
3. Dong H-J, Wressle E, Marcusson J: **Multimorbidity patterns of and use of health services by Swedish 85-year-olds: an exploratory study**. *BMC Geriatrics* 2013, **13**(1):120-120.
4. Liao C-C, Li C-R, Lee S-H, Liao W-C, Liao M-Y, Lin J, Yeh C-J, Lee M-C: **Social support and mortality among the aged people with major diseases or ADL disabilities in Taiwan: A national study**. *Archives of Gerontology & Geriatrics* 2015, **60**(2):317-321.
5. McGilton KS, Heath H, Chu CH, Bostrom AM, Mueller C, Boscart VM, McKenzie-Green B, Moghabghab R, Bowers B: **Moving the agenda forward: a person-centred framework in long-term care**. *Int J Older People Nurs* 2012, **7**(4):303-309.
6. Winefield H. MT, Clifford J., Farmer E.: **The search for reliable and valid measures of patient-centredness.** *Psychol Health* 1996(11):811-824.
7. Edvardsson DW, B. Sandman, P O.: **Person-centred care of people with severe Alzheimer's disease: current status and ways forward**. *Lancet Neurology* 2008(4):362.
8. McCormack B: **Person-centredness in gerontological nursing: an overview of the literature**. *J Clin Nurs* 2004, **13**(3a):31-38.
9. McCance T, Slater P, McCormack B: **Using the caring dimensions inventory as an indicator of person-centred nursing**. *Journal of Clinical Nursing* 2009, **18**(3):409-417.
10. Buckley C, McCormack B, Ryan A: **Valuing narrative in the care of older people: A framework of narrative practice for older adult residential care settings**. *Journal of Clinical Nursing* 2014, **23**(17-18):2565-2577.
11. Edvardsson D, Fetherstonhaugh D, Nay R: **Promoting a continuation of self and normality: person-centred care as described by people with dementia, their family members and aged care staff**. *Journal of Clinical Nursing* 2010, **19**(17-18):2611-2618.
12. McCormack B, Dewing J, Breslin L, Coyne-Nevin A, Kennedy K, Manning M, Peelo-Kilroe L, Tobin C, Slater P: **Developing person-centred practice: nursing outcomes arising from changes to the care environment in residential settings for older people**. *Int J Older People Nurs* 2010, **5**(2):93-107.
13. Crandall LG, White DL, Schuldheis S, Talerico KA: **Initiating person-centered care practices in long-term care facilities**. *Journal of Gerontological Nursing* 2007, **33**(11):47-56.
14. McCormack B, McCance TV: **Development of a framework for person-centred nursing**. *Journal of Advanced Nursing* 2006, **56**(5):472-479.

For peer review only - http://bmjopen.bmj.com/site/about/guidelines.xhtml

For peer review only

15. Edvardsson D, Fetherstonhaugh D, McAuliffe L, Nay R, Chenco C: **Job satisfaction amongst aged care staff: exploring the influence of person-centered care provision**. *Int Psychogeriatr* 2011, **23**(8):1205-1212.
16. McKeown J, Clarke A, Ingleton C, Ryan T, Repper J: **The use of life story work with people with dementia to enhance person-centred care**. *International Journal of Older People Nursing* 2010, **5**(2):148-158.
17. Bone C, Cheung G, Wade B: **Evaluating person centred care and Dementia Care Mapping in a psychogeriatric hospital in New Zealand: a pilot study**. *New Zealand Journal of Occupational Therapy* 2010, **57**(1):35-40.
18. Edvardsson D, Sandman PO, Rasmussen B: **Construction and psychometric evaluation of the Swedish language Person-centred Climate Questionnaire - staff version**. *J Nurs Manag* 2009, **17**(7):790-795.
19. Edvardsson D, Sjogren K, Lindkvist M, Taylor M, Edvardsson K, Sandman PO: **Person-centred climate questionnaire (PCQ-S): establishing reliability and cut-off scores in residential aged care**. *J Nurs Manag* 2015, **23**(3):315-323.
20. Edvardsson D, Koch S, Nay R: **Psychometric evaluation of the English language Person-centred Climate Questionnaire--staff version**. *J Nurs Manag* 2010, **18**(1):54-60.
21. Guillemin F: **Cross-cultural adaptation and validation of health status measures**. *Scand J Rheumatol* 1995, **24**(2):61-63.
22. Beaton DE, Bombardier C, Guillemin F, Ferraz MB: **Guidelines for the process of cross-cultural adaptation of self-report measures**. *Spine (Phila Pa 1976)* 2000, **25**(24):3186-3191.
23. Bland JM, Altman DG: **Cronbach's alpha**. *BMJ* 1997, **314**(7080):572.
24. Landis JR, Koch GG: **The measurement of observer agreement for categorical data**. *Biometrics* 1977, **33**(1):159-174.
25. Bergland A, Kirkevold M, Edvardsson D: **Psychometric properties of the Norwegian Person-centred Climate Questionnaire from a nursing home context**. *Scand J Caring Sci* 2012, **26**(4):820-828.

For peer review only

For peer review only - http://bmjopen.bmj.com/site/about/guidelines.xhtml

Table 1 Demographic characteristics of the study sample (n=163)

Characteristics	n (%)
Gender	
Female	151 (92.6)
Male	12 (7.4)
Age (years)	
18-30	95 (58.3)
31-39	33 (20.2)
⩾40	35 (21.5)
Level of education	
High school	7 (4.3)
Secondary school	37 (22.7)
Junior college	65 (39.9)
Bachelor or higher	54 (33.2)
Ethnicity	
Han	118 (72.4)
Minorities	45 (27.6)
Healthcare staff	
Registered nurse	101 (62.0)
Enrolled nurse	29 (17.8)
Physician	33 (20.2)

For peer review only

Table 2 Rotated component matrix for PCA of the Chinese PCQ-S (n=163)

Item number	Item content	Factor loadings		
		Subscale 1: A climate of safety	Subscale 2: A climate of everydayness	Subscale 3: A climate of community
1	A place where I feel welcome	0.83		
2	A place where I feel acknowledged as a person	0.84		
3	A place where I feel I can be myself	0.58		
4	A place where the patients are in safe hands	0.66		
5	A place where the staff use a language that the patients can understand	0.60		
6	A place which feels homely even though it is in an institution		0.82	
7	A place where there is something nice to look at		0.81	
8	A place where it is quiet and peaceful		0.78	
9	A place where it is possible to get unpleasant thoughts out of your head		0.74	
10	A place which is neat and clean		0.68	
11	A place where it is easy for the patients to keep in contact with their loved ones			0.64
12	A place where it is easy for the patients to receive visitors			0.87
13	A place where it is easy for the patients to talk to the staff			0.85
14	A place where the patients have someone to talk to if they so wish			0.66
Total variance explained (%)	73.3 (total 3 subscales)	55.6	9.5	8.2
Cronbach's alpha	0.94 (total 14 items)	0.87	0.90	0.88

For peer review only - http://bmjopen.bmj.com/site/about/guidelines.xhtml

Table 3 Item performance and reliability test of the Chinese PCQ-S (n=163)

Item number	Item content	Mean±SD	Corrected item-total correction	Cronbach's alpha if item deleted
1	A place where I feel welcome	4.04±0.93	0.62	0.93
2	A place where I feel acknowledged as a person	4.07±0.92	0.54	0.93
3	A place where I feel I can be myself	3.58±1.32	0.70	0.93
4	A place where the patients are in safe hands	4.06±0.96	0.72	0.93
5	A place where the staff use a language that the patients can understand	3.90±1.01	0.72	0.93
6	A place which feels homely even though it is in an institution	3.80±1.13	0.77	0.93
7	A place where there is something nice to look at	3.60±1.15	0.76	0.93
8	A place where it is quiet and peaceful	3.80±1.04	0.78	0.93
9	A place where it is possible to get unpleasant thoughts out of your head	3.20±1.34	0.66	0.93
10	A place which is neat and clean	3.85±1.01	0.70	0.93
11	A place where it is easy for the patients to keep in contact with their loved ones	3.88±1.03	0.79	0.93
12	A place where it is easy for the patients to receive visitors	3.40±1.36	0.59	0.93
13	A place where it is easy for the patients to talk to the staff	3.72±1.16	0.71	0.93
14	A place where the patients have someone to talk to if they so wish	3.94±1.03	0.67	0.93

For peer review only - http://bmjopen.bmj.com/site/about/guidelines.xhtml

Table 4 Test-retest reliability of the Chinese PCQ-S (n=163)

Scale dimension	1st test (Mean±SD)	2nd test (Mean±SD)	P	Pearson correlation coefficient (r)	ICC (95% CI)
A climate of safety	19.7±4.2	19.8±4.0	0.30	0.88	0.93 (0.91, 0.95)
A climate of everydayness	18.3±4.8	18.1±4.9	0.38	0.91	0.95 (0.93, 0.96)
A climate of community	15.0±4.0	14.7±4.1	0.18	0.79	0.92 (0.89, 0.94)
Overall scale	52.9±11.4	52.6±11.7	0.40	0.93	0.97 (0.95-0.98)

For peer review only

For peer review only - http://bmjopen.bmj.com/site/about/guidelines.xhtml

BMJ Open

Psychometric evaluation of the Chinese version of the Person-centred Climate Questionnaire for staff

Journal:	*BMJ Open*
Manuscript ID	bmjopen-2017-017250.R1
Article Type:	Research
Date Submitted by the Author:	12-Jun-2017

Complete List of Authors:	Cai, Le; School of Public Health<br>Ahlström, Gerd; Lunds Universitet<br>Tang, Pingfen; Kunming Medical University<br>Ma, Ke; The third people's hospital of Kunming<br>Edvardsson, David; La Trobe University School of Nursing and Midwifery<br>Behm, Lina; Lunds Universitet<br>Fu, Haiyan; the third people's hospital of Kunming<br>Zhang, Jie; the third people's hospital of Kunming<br>Yang, Jiqun; the third people's hospital of Kunming

**Primary Subject Heading**:	Nursing
Secondary Subject Heading:	Geriatric medicine, Palliative care, Patient-centred medicine
Keywords:	GERIATRIC MEDICINE, PALLIATIVE CARE, Person-centred care, Psychometric evaluation, China, Nursing staff

SCHOLARONE™ Manuscripts

Psychometric evaluation of the Chinese version of the Person-centred Climate Questionnaire for staff

Cai Le [1], Gerd Ahlström [2], Tang Pingfen [1], Ma Ke [3], David Edvardsson [4], Lina Behm [2], Fu Haiyan [3], Zhang Jie [3] and Yang Jiqun [3]

Address: [1] 1168 Yu Hua Street Chun Rong Road, Cheng Gong New City, School of Public Health, Kunming Medical University, Kunming 650500, China;

[2] Department of Health Sciences, Faculty of Medicine, Lund University, Sweden;

[3] Division of palliative care, the third people's hospital of Kunming, China;

[4] Austin Health/Northern Health Clinical Schools of Nursing College of Science, Health and Engineering, La Trobe University, Australia

Email address:

Cai Le * : 1018606825@qq.com;

Gerd Ahlström: gerd.ahlstrom@med.lu.se;

Tang Pingfen: yangyi57@vip.126.com;

Ma Ke: kmmake@126.com;

David Edvardsson: D.Edvardsson@latrobe.edu.au;

Lina Behm: Lina.behm@med.lu.se;

Fu Haiyan: fuhaiyan0622@163.com;

Zhang Jie: 13888437137@126.com;

Yang Jiqun: 1181780508@qq.com

* Corresponding author

Cai Le, Address: 1168 Yu Hua Street Chun Rong Road, Cheng Gong New City, School of Public Health, Kunming Medical University, Kunming 650500, China, Email: 1018606825@qq.com, Phone : 86-871-65922915

Word count 3124

For peer review only

Psychometric evaluation of the Chinese version of the Person-centred Climate Questionnaire for staff

Abstract

Objectives

The aim of the study was to conduct a cross-cultural adaptation of the English version of the Person-centred Climate Questionnaire – staff version (PCQ-S) — for Chinese palliative care staff and to evaluate the psychometric properties of the translated Chinese version in a hospital context.

Design

This was a cross-sectional design. The 14-item English PCQ-S was translated and backtranslated using established procedures. Construct validity and reliability including internal consistency and test-retest reliability were assessed among hospital staffs. Construct validity was tested using Principal component analysis (PCA). Internal consistency was tested using Cronbach's alpha. Test-retest reliability was evaluated through the Pearson correlation coefficient (r) and intra-class correlation coefficient (ICC).

Setting

This study was conducted in three hospitals in Kunming, the capital of the Yunnan province in south-west China.

Participants

A sample of hospital staff (n=163) on duty at the departments of palliative care in three hospitals of Kunming consented to participate in the study.

Results

The results confirmed that the 14-item Chinese PCQ-S consisted of the three subscales shown in other language versions. It showed strong internal consistency through a Cronbach's alpha of 0.94 for the total scale, 0.87 for the safety subscale, 0.90 for the everydayness subscale and 0.88 for the community subscale. The Chinese

For peer review only - http://bmjopen.bmj.com/site/about/guidelines.xhtml

PCQ-S had high test-retest reliability as evidenced by a high correlation coefficient on all scale levels between test and retest scores, on 'a climate of safety' ($r = 0.88$, $P<0.01$), 'a climate of everydayness' ($r=0.91$, $P<0.01$), 'a climate of community' ($r=0.79$, $P<0.01$) and on overall scale scores ($r=0.93$, $P<0.01$). The ICC to evaluate the test-retest reliability was 0.97 (95% CI: 0.95- 0.98).

Conclusions

The Chinese version of PCQ-S is a seemingly valid and reliable tool, which showed satisfactory reliability and validity for assessing staff perceptions of the person-centredness climate in Chinese hospital environments.

Keywords: Geriatric medicine; China; Nursing staff; Palliative care; Person-centred care; Psychometric evaluation

Article summary

Strengths and limitations of this study

- The first study to validate the PCQ-S in an Asian population.
- High response rate (90%) in this study.
- Convenience sampling method may limit the ability to generalize the results.
- The Chinese PCQ-S has been tested only in this hospital palliative care environment.

For peer review only

For peer review only - http://bmjopen.bmj.com/site/about/guidelines.xhtml

INTRODUCTION

Population aging is a global phenomenon and has become a significant public health problem worldwide. Along with the high-speed economic growth and demographic change in the last two decades, China is becoming one of the most rapidly ageing countries in the world. The proportion of older people aged 60 years or more was 13.3% in 2010[1], and is projected to reach 32.8% by 2050[2]. Studies have indicated that older people are more likely to suffer from various diseases, and more particularly chronic disease and comorbidity, which is more difficult to cure due to complexity and coexistence[3]. Chronic diseases may result in disability in older people; therefore, the rising number of older people increases the demand for hospitalization and special care and support from multiple care professionals and providers[4]. This presents a key challenge for Chinese healthcare systems to provide high-quality care for this group. Rapid population ageing in China is also increasing the numbers of older people who are likely to require palliative care in response to higher levels of poor health and chronic diseases. However, palliative care in China has developed more slowly than in high-income Western countries. Palliative care is rooted in harmony between mind and body in traditional Chinese medicine (TCM) with its long history developed over a few thousand years[5, 6].

In recent decades, person-centred care (PCC) has become recognized as a quality focus of elderly care services, which emphasizes the individual's perspective and active participation in the care process[7]. PCC as a concept implies in various ways assisting an individual to be able to be a "whole" human being, by encouraging them to participate in decisions and adjusting the physical environment and the content of the care to fit the needs of each individual. It is defined as 'valuing people as individuals' in delivering health care[8] and is based on people's subjective experience of illness instead of the disease[9-11]. The care process becomes the foundation for how PCC should be provided and the relationship between the professional caregiver and the care recipient becomes essential[12-14].

For peer review only

Person-centredness is now regarded as a central feature of high-quality long-term care for older persons. As such PCC must be a part of the care organisation priorities, and the system needs to support and sustain this change through policy and procedures, job descriptions and education[15]. PCC improves autonomy in elderly care through its focus on individual care plans and support for next of kin, who are seen as important resources[16]. There is evidence to indicate that the person-centredness of a setting is associated with staff's satisfaction with work[17], and staff perceptions of and relationships with patients are crucially important to quality care. Also, for the older person, a person-centered setting has been shown to increase wellbeing and decrease discomfort[18, 19].

Internationally, various instruments have been developed to evaluate the PCC perspectives of professionals who work in elderly care facilities, which include the Person-centred Climate Questionnaire — staff version (PCQ-S)[20], the Person-centered Care Assessment Tool (P-CAT)[21], the Staff Assessment Person Directed Care (PDC)[22], the Individualized Care (IC)[23], and the Staff Person-Centred Practices in Assisted Living (Staff PC-PAL)[24].

Edvardsson and colleagues developed the Swedish-language Person-centred Climate Questionnaire – staff version (PCQ-S) — for evaluating to what extent the climate of care environments is experienced as being person-centred by staff[20, 25]. The questionnaire comprises three subscales (safety, everydayness, and community). It has been validated with older persons being cared for in hospitals, and shown to have satisfactory psychometric properties, which has a total Cronbach's alpha of 0.88 and values of 0.84, 0.80, 0.77 respectively for the three subscales, and satisfactory test–retest reliability, which showed an average measure intra-class correlation coefficient of 0.51 with a 95% confidence interval ranging from 0.47 to 0.75. It is one of the most commonly used instruments internationally. This instrument has been translated from Swedish into Norwegian[26], English[27] and Slovenian[28]. Both the original and the translated Norwegian, English and Slovenian scales have been demonstrated to be

For peer review only - http://bmjopen.bmj.com/site/about/guidelines.xhtml

valid and reliable tools for assessing staff perceptions of person-centredness. However, there exists no Chinese version of the PCQ-S so far, which presents a barrier to measuring and developing person-centred care and to conducting further studies in China with possibilities of making international comparisons. We believe that among the existing tools, the English PCQ-S was the most favourable one for adaptation to the Chinese context due to the rigorous theoretical underpinnings deriving from the perception of person-centredness as emerging from experiences of the whole caring environment, which rings true in Chinese culture as well. So the purpose of this study was to conduct a cross-cultural adaptation of the English version of PCQ-S for Chinese health care staff and to evaluate the psychometric properties of the translated Chinese version in a hospital palliative care context.

METHODS

Instrument

The English PCQ-S questionnaire consists of 14 items and has three subscales (a climate of safety, everydayness, and community)[25]. A climate of safety is measured through items 1-5, everydayness is measured through items 6-10, and community is measured through items 11-14. Scoring is performed on a 6-point Likert-type scale, ranging from 0 (No, I disagree completely) to 5 (Yes, I agree completely). Aggregated scores are calculated using simple sum scores at subscale and total scale levels, which range from 0 to 70, with higher scores indicating a setting perceived as being more person-centred. The English PCQ-S has previously been used and tested in hospital settings, and demonstrated to be a valid and reliable tool for assessing staff perceptions of the unit's person-centredness[27].

Translation and cross-cultural adaptation of the PCQ-S

The translation and cross-cultural adaptation was carried out according to previously published international test commission guidelines[29, 30]. First, forward translation from English to Chinese was performed independently by three native Chinese, two of them university graduates with Public Health background and the other a physician

For peer review only - http://bmjopen.bmj.com/site/about/guidelines.xhtml

familiar with palliative care. A consensus version was obtained after a discussion between the three translators. Second, the consensus version was back-translated into English by two bilingual translators blinded to the procedures of the forward translation. However, the back-translated version was not discussed with the authors of the English-language version of the PCQ-S. Finally, a thorough comparison of the original, translated and back-translated versions was conducted by an expert committee, which consisted of all translators, three palliative care physicians and two university professors. Discrepancies in translations were discussed and resolved, a few wordings were adapted to the Chinese cultural setting, and a consensus pre-final version was established. A final Chinese version was generated after pre-testing through face-validity the pre-final version on 10 staff from a municipal hospital in Kunming. No changes were made after the pre-testing. The 10 staff participating in face-validity of the pre-final version did not subsequently take part in the study itself.

Sample and participants

Three municipal hospitals in Kunming, the capital of Yunnan province in south-west China, were selected through a convenience sampling method. The following inclusion criteria were used: hospital at municipal level located in Kunming city, having department of palliative care in hospital. Their participation was approved by the hospital directors. All staff (n=182) on duty (both morning and afternoon shifts on one specific day) at the departments of palliative care in these three hospitals were considered eligible for participation, and invited to complete the Chinese PCQ-S questionnaire. The sample size in our study was in accordance with the criteria proposed by Terwee et al.[31]. The eligible staff received both oral and written information about the study. Before data collection, each participant was given a full explanation of the research purpose, and was informed that they were under no obligation to participate in the study, and were allowed to withdraw from the study at any time without any prejudice or repercussions. Finally,163 agreed to participate, representing an overall response rate of 90%. The participants completed questionnaires for both the test and retest assessments.

For peer review only - http://bmjopen.bmj.com/site/about/guidelines.xhtml

Data collection

Demographic data was collected along with the questionnaire survey including staff age, gender, level of education, duration of work experience, ethnicity and health care staff position. Each participant was assigned a number by the data collector to indicate his or her identity, so they were anonymous with regard to completing the questionnaire. Two university graduates distributed questionnaires to all appointed staff, and completed questionnaires were anonymously collected on site. To examine the test-retest reliability, all participants were asked to complete the same PCQ-S questionnaire 1 week later. Persons who were not available then were invited to complete the PCQ-S questionnaire on another day as close as possible to the 1-week post-test completion. Data were collected during October and November 2016.

Psychometric evaluation

No variable had missing values. All complete data were included in the analysis. Construct validity was estimated using exploratory factor analysis (principal component analysis (PCA)) with both varimax orthogonal and oblique orthogonal rotation, and goodness-of-fit through confirmative factor analysis[32]. The analysis indicated no difference between the two methods, so only the results from the analysis with varimax orthogonal rotation were presented.

Bartlett's test of sphericity was used to assess whether the correlation between items was adequate based on a criterion of $p < 0.0001$. The Kaiser-Meyer-Olkin (KMO) statistic was used to measure sample adequacy based on a criterion of ≥ 0.7. Principal components were extracted when Kaiser's criterion of eigenvalues was ≥ 1. A component loading cut off of 0.5 was used to decide if an item loaded on a specific component[33]. PCA with oblique rotation was performed to ensure independence of the items.

Reliability testing included assessments of internal consistency and test-retest

For peer review only

reliability. Internal consistency for total and subscale scores was estimated using the Cronbach's alpha coefficient, and the cut-off scores for acceptable reliability were set to item-total correlations of ≥0.5 and in such a way that the Cronbach's alpha would not be increased by item deletion[34]. A Cronbach's alpha between >0.8 and > 0.95 was taken to indicate that the questionnaire had good or excellent internal consistency[34]. Test–retest reliability was evaluated through the Pearson correlation coefficient (r) and a single measure two-way mixed effects model intra-class correlation (ICC), where an ICC >0.80 was taken to indicate satisfactory reliability[35]. The paired t-test was used to determine whether mean scores of the test and retest questionnaires differed significantly. All statistical significance decisions were based on two-tailed P values of <0.05. All data analyses were conducted using the SPSS 17.0 software.

RESULTS

Demographic characteristics of the study group

Table 1 shows the demographic characteristics of the study group. The sample consisted of 92.6% female and 7.4% male staff. The mean age was 31.6 years (SD ± 10.1), with an average length of work experience in healthcare of 8.1 years (SD ± 7.4). The percentage of ethnic minorities was more than one fourth. Most participants were registered nurses (62.0%) or enrolled nurses (17.8%). About one third (33.2%) of the participants had a Bachelor's degree or higher (see Table 1).

Construct validity

The results of the PCA with Bartlett's test (p< 0.0001) and the KMO Measure (0.91) indicated that correlations between items were sufficiently large to perform the PCA. Only the first three components had eigenvalues greater than one, explaining 73.3% of the total variance. Therefore, the PCA resulted in a three-component rotated solution. As shown in Table 2, the first and the second component consisted of five items (loadings between 0.58 and 0.84 vs. loadings between 0.68 and 0.82), where the first component confirmed the sub-scale 'A climate of safety' and where the second

For peer review only - http://bmjopen.bmj.com/site/about/guidelines.xhtml

component confirmed the sub-scale ‘A climate of everydayness’ in the setting. The third component comprised four items (loadings between 0.64 and 0.87), and confirmed the sub-scale ‘A climate of community’.

The three-component model was also evaluated by confirmative factor analysis, and goodness of fit was estimated using indices of the Root Mean Square Error of Approximation (RMSEA), the Normed Fit Index (NFI) and the Comparative Fit Index (CFI). The results indicated that the goodness of fit of the questionnaire was 0.78 for the RMSEA, 0.91 for the NFI and 0.92 for the CFI. Thus, the confirmatory factor analysis supported the exploratory findings, and the three-component model provided adequate fit indices for the questionnaire.

Reliability

Table 2 shows that the Cronbach’s alpha coefficient of the 14-item Chinese PCQ-S was 0.94 for the total scale, 0.87 for the safety subscale, 0.90 for the everydayness subscale and 0.88 for the community subscale, indicating a strong internal consistency reliability overall. Furthermore, the corrected item-total correlations for all items ranged from 0.54 to 0.79, indicating that each item correlated adequately with the total score and thus that the scale is homogenous without any item being redundant (Table 3).

Table 4 presents the results from the test-retest reliability assessment of the Chinese PCQ-S. According to the Pearson’s correlation coefficient analyses, the Chinese PCQ-S indicated high correlation between test and retest on all scale levels: on the sub-scales ‘a climate of safety’ ($r = 0.88$, $P<0.01$), ‘a climate of everydayness’ ($r=0.91$, $P<0.01$) and ‘a climate of community’ ($r=0.79$, $P<0.01$) as well as on the overall scale scores between test and retest ($r=0.93$, $P<0.01$). A paired t-test also confirmed that there was no significant difference between the mean scores of the PCQ-S at the test and retest ratings ($P>0.05$). The ICC of the total score between the test and retest times was 0.97, providing further support that the scale had satisfactory test–retest

For peer review only - http://bmjopen.bmj.com/site/about/guidelines.xhtml

For peer review only

reliability.

DISCUSSION

This is the first study to validate the PCQ-S in an Asian population, and the results of the present study indicated that the cross-culturally adapted Chinese version PCQ-S showed excellent reliability and validity for evaluating staff perceptions of person-centredness in Chinese hospital contexts, which enables further studies and international comparisons.

In this study, the English PCQ-S was cross-culturally adapted into the Chinese version and showed satisfactory psychometric properties (construct validity, test-retest reliability and internal consistency). During our translation of the English PCQ-S into Chinese, a minor cultural discrepancy was encountered and one item of the PCQ-S was therefore modified accordingly. In this instance, ‘peaceful’ was replaced by ‘harmonious’ as this word is closer to Chinese culture. Construct validity was estimated using PCA with varimax orthogonal rotation, resulting in a stable three-factor solution explaining 73.3% of the total variance. The ICC for the overall Chinese PCQ-S scale was 0.97 and for the three subscales was 0.93, 0.95 and 0.92, demonstrating that the test-retest reliability of the overall scale and different domains was excellent. Furthermore, strong internal consistency of the Chinese PCQ-S was demonstrated, as evidenced through a Cronbach’s alpha of 0.89 for the total scale, 0.87 for the safety subscale, 0.90 for the everydayness subscale and 0.88 for the community subscale.

This Chinese version of the PCQ-S showed the same sub-scale structure as the Swedish, Norwegian and Slovenian versions - a structure with the three subscales described above (a climate of safety, everydayness and community) consisting of 14 items. However, it showed a different structure with the English PCQ-S - a four-component structure (a climate of safety, everydayness, community and comprehensibility) consisting of 14 items, which may reflect a difference in cultural

For peer review only

context. Even though the original English PCQ-S described a slightly different structure consisting of four subscales, the instrument developers have recently suggested keeping to the three subscale structure for scoring and comparison purposes as this confirms the theory on which the scale rests [20].

In the Chinese PCQ-S, the ICC (0.97) and Cronbach's alpha for the total scale (0.94) were much higher than in the Swedish (0.51 vs. 0.88) and English (0.75 vs. 0.89) versions, and the Cronbach's alpha for the total scale was also higher than it was in the Norwegian version (0.92). Due to the larger the sample size in our study, which differs from those in the above three studies, the Chinese PCQ-S may have stronger test-retest reliability and internal consistency compared to other language versions of the PCQ-S. The results demonstrated that the Chinese PCQ-S has good reproducibility and well maintains the properties of the original version, and can thus be used in Chinese hospital environments.

The following limitations of the present study should be noted. First, the study employed a convenience sampling method to select staff in palliative care from public hospitals, which may limit the ability to generalize the results to staff in general at Chinese hospitals or other staff working other health care contexts. Second, the Chinese PCQ-S questionnaire has been tested only in this hospital environment, and further psychometric testing of the scale in other settings like nursing homes would be helpful to enable further rigorous comparisons of Chinese PCQ-S in different contexts and settings. Third, the questionnaire had been translated only from the secondary English version, not from the original Swedish version. Fourth, with respect to the psychometric assessment of the PCQ-S, criterion-related validity, convergent validity and discriminative validity were not taken into account. Further study is needed to explore this in the future. Fifth, the back-translated version was not validated, due to the cross-cultural adaptation.

Conclusion

For peer review only - http://bmjopen.bmj.com/site/about/guidelines.xhtml

The 14-item Chinese PCQ-S is a cross-culturally adapted version of the English PCQ-S, which showed excellent psychometric properties in terms of reliability and validity for evaluating staff perceptions of the person-centredness in Chinese hospital environments. Our results indicated that the Chinese version of the PCQ-S can be utilized for future measurement and development of person-centred care in China and for conducting cross-cultural international comparisons with for example Sweden.

Ethical approval and consent to participate

This study was approved by the Ethics Committee of Kunming Medical University. Oral and written informed consent was obtained from all persons participating in the study.

Availability of data and material

The datasets used and/or analysed during the current study are available from the corresponding author on request.

Competing interests

The authors declare that there are no conflicts of interest.

Consent for publication

Not applicable.

Funding

The study was supported by grants from the National Natural Science Fund of China (Grant number: 81611130077), the Swedish Research Council (Grant number: 2015-06243) and the Major Union Specific Project Foundation of Yunnan Provincial Science and Technology Department and Kunming Medical University (2016).

Authors' contributions

CL (Kunming Medical University, China) was responsible for the study design, data

For peer review only - http://bmjopen.bmj.com/site/about/guidelines.xhtml

analysis, and drafting the paper. GA (Lund University, Sweden), TP (Kunming Medical University, China) and MK (the third people's hospital of Kunming, China) contributed to the study design and provided comments on the paper during the writing process. DE (La Trobe University, Australia) and LB (Lund University, Sweden) provided comments on the paper during the writing process. FH, ZJ and JY (the third people's hospital of Kunming, China) were responsible for the data collection.

Acknowledgements

We would like to thank Ms Yang Jingjing and Ms Wu Chao, two postgraduates of the School of Public Health, Kunming Medical University, who helped us with data collection and Magnus Persson, Lund University, Sweden for providing comments on the paper.

For peer review only

REFERENCES

1. Sixth National Population Census of the People's Republic of China. Beijing, China: National Bureau of Statistics of China 2011.
2. World population prospects: the 2012 revision. New York, United States: Department of Economic and Social Affairs (DoEaSA), Population Division, United Nations 2013.
3. Dong H-J, Wressle E, Marcusson J. Multimorbidity patterns of and use of health services by Swedish 85-year-olds: an exploratory study. *BMC Geriatr* 2013;13(1):120-20. doi: 10.1186/1471-2318-13-120
4. Liao C-C, Li C-R, Lee S-H, et al. Social support and mortality among the aged people with major diseases or ADL disabilities in Taiwan: A national study. *Arch Gerontol Geriatr* 2015;60(2):317-21. doi: 10.1016/j.archger.2014.11.007
5. Hsu CY, O'Connor M, Lee S. Understandings of death and dying for people of Chinese origin. *Death Stud* 2009;33(2):153-74. doi: 10.1080/07481180802440431
6. Chan CLW, Chow AYM. Death, dying and bereavement : a Hong Kong Chinese experience. Hong Kong: Hong Kong University Press 2006.
7. McGilton KS, Heath H, Chu CH, et al. Moving the agenda forward: a person-centred framework in long-term care. *Int J Older People Nurs* 2012;7(4):303-9. doi: 10.1111/opn.12010
8. Winefield H. MT, Clifford J., Farmer E. The search for reliable and valid measures of patient-centredness. *Psychol Health* 1996(11):811-24.
9. Edvardsson DW, B. Sandman, P O. Person-centred care of people with severe Alzheimer's disease: current status and ways forward. *Lancet Neurology* 2008(4):362. doi: 10.1016/S1474-4422(08)70063-2
10. McCormack B. Person-centredness in gerontological nursing: an overview of the literature. *J Clin Nurs* 2004;13(3a):31-8. doi: 10.1111/j.1365-2702.2004.00924.x
11. McCance T, Slater P, McCormack B. Using the caring dimensions inventory as an indicator of person-centred nursing. *J Clin Nurs* 2009;18(3):409-17. doi: 10.1111/j.1365-2702.2008.02466.x
12. Buckley C, McCormack B, Ryan A. Valuing narrative in the care of older people: A framework of narrative practice for older adult residential care settings. *J Clin Nurs* 2014;23(17-18):2565-77. doi: 10.1111/jocn.12472
13. Edvardsson D, Fetherstonhaugh D, Nay R. Promoting a continuation of self and normality: person-centred care as described by people with dementia, their family members and aged care staff. *J Clin Nurs* 2010;19(17-18):2611-18. doi: 10.1111/j.1365-2702.2009.03143.x
14. McCormack B, Dewing J, Breslin L, et al. Developing person-centred practice: nursing outcomes arising from changes to the care environment in residential settings for older people. *Int J Older People Nurs* 2010;5(2):93-107. doi: 10.1111/j.1748-3743.2010.00216.x
15. Crandall LG, White DL, Schuldheis S, et al. Initiating person-centered care practices in long-term care facilities. *J Gerontol Nurs* 2007;33(11):47-56.
16. McCormack B, McCance TV. Development of a framework for person-centred nursing. *J Adv Nurs* 2006;56(5):472-79. doi: 10.1111/j.1365-2648.2006.04042.x
17. Edvardsson D, Fetherstonhaugh D, McAuliffe L, et al. Job satisfaction amongst aged care staff: exploring the influence of person-centered care provision. *Int Psychogeriatr* 2011;23(8):1205-12. doi: 10.1017/S1041610211000159

For peer review only

18. McKeown J, Clarke A, Ingleton C, et al. The use of life story work with people with dementia to enhance person-centred care. *Int J Older People Nurs* 2010;5(2):148-58. doi: 10.1111/j.1748-3743.2010.00219.x

19. Bone C, Cheung G, Wade B. Evaluating person centred care and Dementia Care Mapping in a psychogeriatric hospital in New Zealand: a pilot study. *NZJOT* 2010;57(1):35-40.

20. Edvardsson D, Sandman PO, Rasmussen B. Construction and psychometric evaluation of the Swedish language Person-centred Climate Questionnaire - staff version. *J Nurs Manag* 2009;17(7):790-5. doi: 10.1111/j.1365-2834.2009.01005.x

21. Edvardsson D, Fetherstonhaugh D, Nay R, et al. Development and initial testing of the Person-centered Care Assessment Tool (P-CAT). *Int Psychogeriatr* 2010;22(1):101-8. doi: 10.1017/S1041610209990688

22. White DL, Newton-Curtis L, Lyons KS. Development and initial testing of a measure of person-directed care. *Gerontologist* 2008;48 Spec No 1:114-23.

23. Chappell N, Reid R, Gish J. Staff-based measures of individualized care for persons with dementia in long-term care facilities. *Dementia* 2007;6(4):527-47. doi: doi:10.1177/1471301207084372

24. Zimmerman S, Allen J, Cohen LW, et al. A measure of person-centered practices in assisted living: the PC-PAL. *J Am Med Dir Assoc* 2015;16(2):132-7. doi: 10.1016/j.jamda.2014.07.016

25. Edvardsson D, Sjogren K, Lindkvist M, et al. Person-centred climate questionnaire (PCQ-S): establishing reliability and cut-off scores in residential aged care. *J Nurs Manag* 2015;23(3):315-23. doi: 10.1111/jonm.12132

26. Bergland A, Kirkevold M, Edvardsson D. Psychometric properties of the Norwegian Person-centred Climate Questionnaire from a nursing home context. *Scand J Caring Sci* 2012;26(4):820-8. doi: 10.1111/j.1471-6712.2012.00979.x

27. Edvardsson D, Koch S, Nay R. Psychometric evaluation of the English language Person-centred Climate Questionnaire--staff version. *J Nurs Manag* 2010;18(1):54-60. doi: 10.1111/j.1365-2834.2009.01038.x

28. Vrbnjak D, Pahor D, Povalej Brzan P, et al. Psychometric testing of the Slovenian Person-centred Climate Questionnaire - staff version. *J Nurs Manag* 2017 doi: 10.1111/jonm.12479

29. Guillemin F. Cross-cultural adaptation and validation of health status measures. *Scand J Rheumatol* 1995;24(2):61-3.

30. Beaton DE, Bombardier C, Guillemin F, et al. Guidelines for the process of cross-cultural adaptation of self-report measures. *Spine (Phila Pa 1976)* 2000;25(24):3186-91.

31. Terwee CB, Bot SD, de Boer MR, et al. Quality criteria were proposed for measurement properties of health status questionnaires. *J Clin Epidemiol* 2007;60(1):34-42. doi: 10.1016/j.jclinepi.2006.03.012

32. Nunnally JC, Bernstein IH. Psychometric theory. New York: McGraw-Hill 1994.

33. Pett MA, Lackey NR, Sullivan JJ. Making sense of factor analysis : the use of factor analysis for instrument development in health care research. London: SAGE 2003.

34. Bland JM, Altman DG. Cronbach's alpha. *BMJ* 1997;314(7080):572.

35. Landis JR, Koch GG. The measurement of observer agreement for categorical data. *Biometrics* 1977;33(1):159-74.

For peer review only - http://bmjopen.bmj.com/site/about/guidelines.xhtml

Table 1 Demographic characteristics of the study sample (n=163)

Characteristics	n (%)
Gender	
Female	151 (92.6)
Male	12 (7.4)
Age (years)	
18-30	95 (58.3)
31-39	33 (20.2)
⩾40	35 (21.5)
Level of education	
High school	7 (4.3)
Secondary school	37 (22.7)
Junior college	65 (39.9)
Bachelor or higher	54 (33.2)
Ethnicity	
Han	118 (72.4)
Minorities	45 (27.6)
Healthcare staff	
Registered nurse	101 (62.0)
Enrolled nurse	29 (17.8)
Physician	33 (20.2)

For peer review only

For peer review only - http://bmjopen.bmj.com/site/about/guidelines.xhtml

Table 2 Rotated component matrix for PCA of the Chinese PCQ-S (n=163)

Item number	Item content	Factor loadings		
		Subscale 1: A climate of safety	Subscale 2: A climate of everydayness	Subscale 3: A climate of community
1	A place where I feel welcome	0.83		
2	A place where I feel acknowledged as a person	0.84		
3	A place where I feel I can be myself	0.58		
4	A place where the patients are in safe hands	0.66		
5	A place where the staff use a language that the patients can understand	0.60		
6	A place which feels homely even though it is in an institution		0.82	
7	A place where there is something nice to look at		0.81	
8	A place where it is quiet and peaceful		0.78	
9	A place where it is possible to get unpleasant thoughts out of your head		0.74	
10	A place which is neat and clean		0.68	
11	A place where it is easy for the patients to keep in contact with their loved ones			0.64
12	A place where it is easy for the patients to receive visitors			0.87
13	A place where it is easy for the patients to talk to the staff			0.85
14	A place where the patients have someone to talk to if they so wish			0.66
Total variance explained (%)	73.3 (total 3 subscales)	55.6	9.5	8.2
Cronbach's alpha	0.94 (total 14 items)	0.87	0.90	0.88

For peer review only - http://bmjopen.bmj.com/site/about/guidelines.xhtml

Table 3 Item performance and reliability test of the Chinese PCQ-S (n=163)

Item number	Item content	Mean±SD	Corrected item: total correction	Cronbach's alpha if item deleted
1	A place where I feel welcome	4.04±0.93	0.62	0.93
2	A place where I feel acknowledged as a person	4.07±0.92	0.54	0.93
3	A place where I feel I can be myself	3.58±1.32	0.70	0.93
4	A place where the patients are in safe hands	4.06±0.96	0.72	0.93
5	A place where the staff use a language that the patients can understand	3.90±1.01	0.72	0.93
6	A place which feels homely even though it is in an institution	3.80±1.13	0.77	0.93
7	A place where there is something nice to look at	3.60±1.15	0.76	0.93
8	A place where it is quiet and peaceful	3.80±1.04	0.78	0.93
9	A place where it is possible to get unpleasant thoughts out of your head	3.20±1.34	0.66	0.93
10	A place which is neat and clean	3.85±1.01	0.70	0.93
11	A place where it is easy for the patients to keep in contact with their loved ones	3.88±1.03	0.79	0.93
12	A place where it is easy for the patients to receive visitors	3.40±1.36	0.59	0.93
13	A place where it is easy for the patients to talk to the staff	3.72±1.16	0.71	0.93
14	A place where the patients have someone to talk to if they so wish	3.94±1.03	0.67	0.93

For peer review only - http://bmjopen.bmj.com/site/about/guidelines.xhtml

Table 4 Test-retest reliability of the Chinese PCQ-S (n=163)

Scale dimension	1st test (Mean±SD)	2nd test (Mean±SD)	P	Pearson correlation coefficient (r)	ICC (95% CI)
A climate of safety	19.7±4.2	19.8±4.0	0.30	0.88	0.93 (0.91, 0.95)
A climate of everydayness	18.3±4.8	18.1±4.9	0.38	0.91	0.95 (0.93, 0.96)
A climate of community	15.0±4.0	14.7±4.1	0.18	0.79	0.92 (0.89, 0.94)
Overall scale	52.9±11.4	52.6±11.7	0.40	0.93	0.97 (0.95-0.98)

For peer review only

For peer review only - http://bmjopen.bmj.com/site/about/guidelines.xhtml

STROBE Statement—Checklist of items that should be included in reports of ***cross-sectional studies***

	Item No.	Recommendation	Page No.	Relevant text from manuscript
**Title and abstract**	1	(*a*) Indicate the study's design with a commonly used term in the title or the abstract	0	cross-sectional design
		(*b*) Provide in the abstract an informative and balanced summary of what was done and what was found	1-2	Results, Conclusions
**Introduction**				
Background/rationale	2	Explain the scientific background and rationale for the investigation being reported	3-5	Introduction
Objectives	3	State specific objectives, including any prespecified hypotheses	5	The purpose of this study
**Methods**				
Study design	4	Present key elements of study design early in the paper	5	Methods
Setting	5	Describe the setting, locations, and relevant dates, including periods of recruitment, exposure, follow-up, and data collection	-	Sample and Participants, Data collection
Participants	6	(*a*) *Cohort study*—Give the eligibility criteria, and the sources and methods of selection of participants. Describe methods of follow-up<br>*Case-control study*—Give the eligibility criteria, and the sources and methods of case ascertainment and control selection. Give the rationale for the choice of cases and controls<br>*Cross-sectional study*—Give the eligibility criteria, and the sources and methods of selection of participants	6-7	Sample and Participants, inclusion criteria
		(*b*) *Cohort study*—For matched studies, give matching criteria and number of exposed and unexposed<br>*Case-control study*—For matched studies, give matching criteria and the number of controls per case		
Variables	7	Clearly define all outcomes, exposures, predictors, potential confounders, and effect modifiers. Give diagnostic criteria, if applicable	5-8	Results
Data sources/ measurement	8*	For each variable of interest, give sources of data and details of methods of assessment (measurement). Describe comparability of assessment methods if there is more than one group	5-6	Psychometric evaluation
Bias	9	Describe any efforts to address potential sources of bias	11	limitation
Study size	10	Explain how the study size was arrived at	6	Sample size

Continued on next page

For peer review only - http://bmjopen.bmj.com/site/about/guidelines.xhtml

Quantitative variables	11	Explain how quantitative variables were handled in the analyses. If applicable, describe which groupings were chosen and why	7-8	Psychometric evaluation
Statistical methods	12	(*a*) Describe all statistical methods, including those used to control for confounding	7-8	Psychometric evaluation
		(*b*) Describe any methods used to examine subgroups and interactions		
		(*c*) Explain how missing data were addressed		
		(*d*) *Cohort study*—If applicable, explain how loss to follow-up was addressed<br>*Case-control study*—If applicable, explain how matching of cases and controls was addressed<br>*Cross-sectional study*—If applicable, describe analytical methods taking account of sampling strategy		
		(*e*) Describe any sensitivity analyses		
**Results**				
Participants	13*	(a) Report numbers of individuals at each stage of study—eg numbers potentially eligible, examined for eligibility, confirmed eligible, included in the study, completing follow-up, and analysed	6	All staff (n=182) on duty 163 consented to participate
		(b) Give reasons for non-participation at each stage		
		(c) Consider use of a flow diagram		
Descriptive data	14*	(a) Give characteristics of study participants (eg demographic, clinical, social) and information on exposures and potential confounders	8	Demographic characteristics of the study group
		(b) Indicate number of participants with missing data for each variable of interest		
		(c) *Cohort study*—Summarise follow-up time (eg, average and total amount)		
Outcome data	15*	*Cohort study*—Report numbers of outcome events or summary measures over time		
		Case-control study—Report numbers in each exposure category, or summary measures of exposure		
		Cross-sectional study—Report numbers of outcome events or summary measures	8-9	Results
Main results	16	(*a*) Give unadjusted estimates and, if applicable, confounder-adjusted estimates and their precision (eg, 95% confidence interval). Make clear which confounders were adjusted for and why they were included	8-9	
		(*b*) Report category boundaries when continuous variables were categorized	-	
		(*c*) If relevant, consider translating estimates of relative risk into absolute risk for a meaningful time period	-	

Continued on next page

For peer review only - http://bmjopen.bmj.com/site/about/guidelines.xhtml

Other analyses	17	Report other analyses done—eg analyses of subgroups and interactions, and sensitivity analyses	-	
**Discussion**				
Key results	18	Summarise key results with reference to study objectives	10	Discussion
Limitations	19	Discuss limitations of the study, taking into account sources of potential bias or imprecision. Discuss both direction and magnitude of any potential bias	11	The following limitations of the present study should be noted
Interpretation	20	Give a cautious overall interpretation of results considering objectives, limitations, multiplicity of analyses, results from similar studies, and other relevant evidence	10-11	
Generalisability	21	Discuss the generalisability (external validity) of the study results	11	First, the study employed a convenience sampling method to select staff in palliative care from public hospitals, which may limit the ability to generalize the results to a larger population of staff in Chinese hospitals and beyond.
**Other information**				
Funding	22	Give the source of funding and the role of the funders for the present study and, if applicable, for the original study on which the present article is based	12	Funding

-

*Give information separately for cases and controls in case-control studies and, if applicable, for exposed and unexposed groups in cohort and cross-sectional studies.

Note: An Explanation and Elaboration article discusses each checklist item and gives methodological background and published examples of transparent reporting. The STROBE checklist is best used in conjunction with this article (freely available on the Web sites of PLoS Medicine at http://www.plosmedicine.org/, Annals of Internal Medicine at http://www.annals.org/, and Epidemiology at http://www.epidem.com/). Information on the STROBE Initiative is available at www.strobe-statement.org.

For peer review only

For peer review only - http://bmjopen.bmj.com/site/about/guidelines.xhtml

BMJ Open

Psychometric evaluation of the Chinese version of the Person-centred Climate Questionnaire for staff

Journal:	*BMJ Open*
Manuscript ID	bmjopen-2017-017250.R2
Article Type:	Research
Date Submitted by the Author:	17-Jul-2017
Complete List of Authors:	Cai, Le; School of Public Health<br>Ahlström, Gerd; Lunds Universitet<br>Tang, Pingfen; Kunming Medical University<br>Ma, Ke; The third people's hospital of Kunming<br>Edvardsson, David; La Trobe University School of Nursing and Midwifery<br>Behm, Lina; Lunds Universitet<br>Fu, Haiyan; the third people's hospital of Kunming<br>Zhang, Jie; the third people's hospital of Kunming<br>Yang, Jiqun; the third people's hospital of Kunming
**Primary Subject Heading**:	Nursing
Secondary Subject Heading:	Geriatric medicine, Palliative care, Patient-centred medicine
Keywords:	GERIATRIC MEDICINE, PALLIATIVE CARE, Person-centred care, Psychometric evaluation, China, Nursing staff

SCHOLARONE™ Manuscripts

For peer review only

Psychometric evaluation of the Chinese version of the Person-centred Climate Questionnaire for staff

Cai Le [1], Gerd Ahlström [2], Tang Pingfen [1], Ma Ke [3], David Edvardsson [4], Lina Behm [2], Fu Haiyan [3], Zhang Jie [3] and Yang Jiqun [3]

Address: [1] 1168 Yu Hua Street Chun Rong Road, Cheng Gong New City, School of Public Health, Kunming Medical University, Kunming 650500, China;

[2] Department of Health Sciences, Faculty of Medicine, Lund University, Sweden;

[3] Division of palliative care, the third people's hospital of Kunming, China;

[4]Austin Health/Northern Health Clinical Schools of Nursing College of Science, Health and Engineering, La Trobe University, Australia

Email address:

Cai Le * : 1018606825@qq.com;

Gerd Ahlström: gerd.ahlstrom@med.lu.se;

Tang Pingfen: yangyi57@vip.126.com;

Ma Ke: kmmake@126.com;

David Edvardsson: D.Edvardsson@latrobe.edu.au;

Lina Behm: Lina.behm@med.lu.se;

Fu Haiyan: fuhaiyan0622@163.com;

Zhang Jie: 13888437137@126.com;

Yang Jiqun: 1181780508@qq.com

* Corresponding author

Cai Le, Address: 1168 Yu Hua Street Chun Rong Road, Cheng Gong New City, School of Public Health, Kunming Medical University, Kunming 650500, China, Email: 1018606825@qq.com, Phone : 86-871-65922915

Word count 3124

For peer review only

Psychometric evaluation of the Chinese version of the Person-centred Climate Questionnaire for staff

Abstract

Objectives

The aim of the study was to evaluate the psychometric properties of the English version of the Person-centred Climate Questionnaire – staff version (PCQ-S) — for Chinese palliative care staff in a hospital context.

Design

This was a cross-sectional design. The 14-item English PCQ-S was translated and backtranslated using established procedures. Construct validity and reliability including internal consistency and test-retest reliability were assessed among hospital staffs. Construct validity was tested using Principal component analysis (PCA). Internal consistency was tested using Cronbach's alpha. Test-retest reliability was evaluated through the weighted kappa (Kp), Pearson correlation coefficient (r), and intra-class correlation coefficient (ICC).

Setting

This study was conducted in three hospitals in Kunming, the capital of the Yunnan province in south-west China.

Participants

A sample of hospital staff (n=163) on duty at the departments of palliative care in three hospitals of Kunming consented to participate in the study.

Results

The results confirmed that the 14-item Chinese PCQ-S consisted of the three subscales shown in other language versions. It showed strong internal consistency through a Cronbach's alpha of 0.94 for the total scale, 0.87 for the safety subscale, 0.90 for the everydayness subscale and 0.88 for the community subscale. The Chinese PCQ-S had high test-retest reliability as evidenced by a high Kp coefficient and a high

For peer review only - http://bmjopen.bmj.com/site/about/guidelines.xhtml

correlation coefficient on all scale levels between test and retest scores, on 'a climate of safety' (Kp=0.77, r = 0.88, P<0.01), 'a climate of everydayness' (Kp=0.82, r=0.91, P<0.01), 'a climate of community' (Kp=0.75, r=0.79, P<0.01) and on overall scale scores (Kp=0.85, r=0.93, P<0.01). The ICC to evaluate the test-retest reliability was 0.97 (95% CI: 0.95- 0.98).

Conclusions

The Chinese version of PCQ-S is a seemingly valid and reliable tool, which showed satisfactory reliability and validity for assessing staff perceptions of the person-centredness climate in Chinese hospital environments.

Keywords: Geriatric medicine; China; Nursing staff; Palliative care; Person-centred care; Psychometric evaluation

Article summary

Strengths and limitations of this study

- The first study to validate the PCQ-S in an Asian population.
- High response rate (90%) in this study.
- Convenience sampling method may limit the ability to generalize the results.
- The Chinese PCQ-S has been tested only in this hospital palliative care environment.

For peer review only - http://bmjopen.bmj.com/site/about/guidelines.xhtml

INTRODUCTION

Population aging is a global phenomenon and has become a significant public health problem worldwide. Along with the high-speed economic growth and demographic change in the last two decades, China is becoming one of the most rapidly ageing countries in the world. The proportion of older people aged 60 years or more was 13.3% in 2010[1], and is projected to reach 32.8% by 2050[2]. Studies have indicated that older people are more likely to suffer from various diseases, and more particularly chronic disease and comorbidity, which is more difficult to cure due to complexity and coexistence[3]. Chronic diseases may result in disability in older people; therefore, the rising number of older people increases the demand for hospitalization and special care and support from multiple care professionals and providers[4]. This presents a key challenge for Chinese healthcare systems to provide high-quality care for this group. Rapid population ageing in China is also increasing the numbers of older people who are likely to require palliative care in response to higher levels of poor health and chronic diseases. However, palliative care in China has developed more slowly than in high-income Western countries. Palliative care is rooted in harmony between mind and body in traditional Chinese medicine (TCM) with its long history developed over a few thousand years[5, 6].

In recent decades, person-centred care (PCC) has become recognized as a quality focus of elderly care services, which emphasizes the individual's perspective and active participation in the care process[7]. PCC as a concept implies in various ways assisting an individual to be able to be a "whole" human being, by encouraging them to participate in decisions and adjusting the physical environment and the content of the care to fit the needs of each individual. It is defined as 'valuing people as individuals' in delivering health care[8] and is based on people's subjective experience of illness instead of the disease[9-11]. The care process becomes the foundation for how PCC should be provided and the relationship between the professional caregiver and the care recipient becomes essential[12-14].

For peer review only - http://bmjopen.bmj.com/site/about/guidelines.xhtml

Person-centredness is now regarded as a central feature of high-quality long-term care for older persons. As such PCC must be a part of the care organisation priorities, and the system needs to support and sustain this change through policy and procedures, job descriptions and education[15]. PCC improves autonomy in elderly care through its focus on individual care plans and support for next of kin, who are seen as important resources[16]. There is evidence to indicate that the person-centredness of a setting is associated with staff's satisfaction with work[17], and staff perceptions of and relationships with patients are crucially important to quality care. Also, for the older person, a person-centered setting has been shown to increase wellbeing and decrease discomfort[18, 19].

Internationally, various instruments have been developed to evaluate the PCC perspectives of professionals who work in elderly care facilities, which include the Person-centred Climate Questionnaire — staff version (PCQ-S)[20], the Person-centered Care Assessment Tool (P-CAT)[21], the Staff Assessment Person Directed Care (PDC)[22], the Individualized Care (IC)[23], and the Staff Person-Centred Practices in Assisted Living (Staff PC-PAL)[24].

Edvardsson and colleagues developed the Swedish-language Person-centred Climate Questionnaire – staff version (PCQ-S) — for evaluating to what extent the climate of care environments is experienced as being person-centred by staff[20, 25]. The questionnaire comprises three subscales (safety, everydayness, and community). It has been validated with older persons being cared for in hospitals, and shown to have satisfactory psychometric properties, which has a total Cronbach's alpha of 0.88 and values of 0.84, 0.80, 0.77 respectively for the three subscales, and satisfactory test–retest reliability, which showed an average measure intra-class correlation coefficient of 0.51 with a 95% confidence interval ranging from 0.47 to 0.75. It is one of the most commonly used instruments internationally. This instrument has been translated from Swedish into Norwegian[26] and English[27], and the English version has also been translated into Slovenian[28]. Both the original and the translated

For peer review only - http://bmjopen.bmj.com/site/about/guidelines.xhtml

Norwegian, English and Slovenian scales have been demonstrated to be valid and reliable tools for assessing staff perceptions of person-centredness. However, there exists no Chinese version of the PCQ-S so far, which presents a barrier to measuring and developing person-centred care and to conducting further studies in China with possibilities of making international comparisons. We believe that among the existing tools, the English PCQ-S was the most favourable one for adaptation to the Chinese context due to the rigorous theoretical underpinnings deriving from the perception of person-centredness as emerging from experiences of the whole caring environment, which rings true in Chinese culture as well. So the purpose of this study was to conduct a cross-cultural adaptation of the English version of PCQ-S for Chinese health care staff and to evaluate the psychometric properties of the translated Chinese version in a hospital palliative care context.

METHODS

Instrument

The English PCQ-S questionnaire consists of 14 items and has three subscales (a climate of safety, everydayness, and community)[25]. A climate of safety is measured through items 1-5, everydayness is measured through items 6-10, and community is measured through items 11-14. Scoring is performed on a 6-point Likert-type scale, ranging from 0 (No, I disagree completely) to 5 (Yes, I agree completely). Aggregated scores are calculated using simple sum scores at subscale and total scale levels, which range from 0 to 70, with higher scores indicating a setting perceived as being more person-centred. The English PCQ-S has previously been used and tested in hospital settings, and demonstrated to be a valid and reliable tool for assessing staff perceptions of the unit's person-centredness[27].

Translation and cross-cultural adaptation of the PCQ-S

The translation and cross-cultural adaptation was carried out according to previously published international test commission guidelines[29, 30]. First, forward translation from English to Chinese was performed independently by three native Chinese, two

For peer review only

of them university graduates with Public Health background and the other a physician familiar with palliative care. A consensus version was obtained after a discussion between the three translators. Second, the consensus version was back-translated into English by two bilingual translators blinded to the procedures of the forward translation. However, the back-translated version was not discussed with the authors of the English-language version of the PCQ-S. Finally, a thorough comparison of the original, translated and back-translated versions was conducted by an expert committee, which consisted of all translators, three palliative care physicians and two university professors. Discrepancies in translations were discussed and resolved, a few wordings were adapted to the Chinese cultural setting, and a consensus pre-final version was established. A final Chinese version was generated after pre-testing through face-validity the pre-final version on 10 staff from a municipal hospital in Kunming. No changes were made after the pre-testing. The 10 staff participating in face-validity of the pre-final version did not subsequently take part in the study itself.

Sample and participants

Three municipal hospitals in Kunming, the capital of Yunnan province in south-west China, were selected through a convenience sampling method. The following inclusion criteria were used: hospital at municipal level located in Kunming city, having department of palliative care in hospital. Their participation was approved by the hospital directors. All staff (n=182) on duty (both morning and afternoon shifts on one specific day) at the departments of palliative care in these three hospitals were considered eligible for participation, and invited to complete the Chinese PCQ-S questionnaire. The sample size in our study was in accordance with the criteria proposed by Terwee et al.[31]. The eligible staff received both oral and written information about the study. Before data collection, each participant was given a full explanation of the research purpose, and was informed that they were under no obligation to participate in the study, and were allowed to withdraw from the study at any time without any prejudice or repercussions. Finally,163 agreed to participate, representing an overall response rate of 90%. The participants completed

For peer review only - http://bmjopen.bmj.com/site/about/guidelines.xhtml

questionnaires for both the test and retest assessments.

Data collection

Demographic data was collected along with the questionnaire survey including staff age, gender, level of education, duration of work experience, ethnicity and health care staff position. Each participant was assigned a number by the data collector to indicate his or her identity, so they were anonymous with regard to completing the questionnaire. Two university graduates distributed questionnaires to all appointed staff, and completed questionnaires were anonymously collected on site. To examine the test-retest reliability, all participants were asked to complete the same PCQ-S questionnaire 1 week later. Persons who were not available then were invited to complete the PCQ-S questionnaire on another day as close as possible to the 1-week post-test completion. Data were collected during October and November 2016.

Psychometric evaluation

No variable had missing values. All complete data were included in the analysis. Construct validity was estimated using exploratory factor analysis (principal component analysis (PCA)) with both varimax orthogonal and oblique orthogonal rotation, and goodness-of-fit through confirmative factor analysis[32]. The analysis indicated no difference between the two methods, so only the results from the analysis with varimax orthogonal rotation were presented.

Bartlett's test of sphericity was used to assess whether the correlation between items was adequate based on a criterion of $p < 0.0001$. The Kaiser-Meyer-Olkin (KMO) statistic was used to measure sample adequacy based on a criterion of ≥ 0.7. Principal components were extracted when Kaiser's criterion of eigenvalues was ≥ 1. A component loading cut off of 0.5 was used to decide if an item loaded on a specific component[33]. PCA with oblique rotation was performed to ensure independence of the items.

For peer review only - http://bmjopen.bmj.com/site/about/guidelines.xhtml

Reliability testing included assessments of internal consistency and test-retest reliability. Internal consistency for total and subscale scores was estimated using the Cronbach's alpha coefficient, and the cut-off scores for acceptable reliability were set to item-total correlations of ≥0.5 and in such a way that the Cronbach's alpha would not be increased by item deletion[34]. A Cronbach's alpha between >0.8 and > 0.95 was taken to indicate that the questionnaire had good or excellent internal consistency[34]. Test–retest reliability was evaluated through the weighted kappa coefficient (Kp), Pearson correlation coefficient (r) and a single measure two-way mixed effects model intra-class correlation (ICC), where an ICC >0.80 was taken to indicate satisfactory reliability[35]. The paired t-test was used to determine whether mean scores of the test and retest questionnaires differed significantly. All statistical significance decisions were based on two-tailed P values of <0.05. All data analyses were conducted using the SPSS 17.0 software.

RESULTS

Demographic characteristics of the study group

Table 1 shows the demographic characteristics of the study group. The sample consisted of 92.6% female and 7.4% male staff. The mean age was 31.6 years (SD ± 10.1), with an average length of work experience in healthcare of 8.1 years (SD ± 7.4). The percentage of ethnic minorities was more than one fourth. Most participants were registered nurses (62.0%) or enrolled nurses (17.8%). About one third (33.2%) of the participants had a Bachelor's degree or higher (see Table 1).

Construct validity

The results of the PCA with Bartlett's test (p< 0.0001) and the KMO Measure (0.91) indicated that correlations between items were sufficiently large to perform the PCA. Only the first three components had eigenvalues greater than one, explaining 73.3% of the total variance. Therefore, the PCA resulted in a three-component rotated solution. As shown in Table 2, the first and the second component consisted of five items (loadings between 0.58 and 0.84 vs. loadings between 0.68 and 0.82), where the

For peer review only - http://bmjopen.bmj.com/site/about/guidelines.xhtml

first component confirmed the sub-scale 'A climate of safety' and where the second component confirmed the sub-scale 'A climate of everydayness' in the setting. The third component comprised four items (loadings between 0.64 and 0.87), and confirmed the sub-scale 'A climate of community'.

The three-component model was also evaluated by confirmative factor analysis, and goodness of fit was estimated using indices of the Root Mean Square Error of Approximation (RMSEA), the Normed Fit Index (NFI) and the Comparative Fit Index (CFI). The results indicated that the goodness of fit of the questionnaire was 0.78 for the RMSEA, 0.91 for the NFI and 0.92 for the CFI. Thus, the confirmatory factor analysis supported the exploratory findings, and the three-component model provided adequate fit indices for the questionnaire.

Reliability

Table 2 shows that the Cronbach's alpha coefficient of the 14-item Chinese PCQ-S was 0.94 for the total scale, 0.87 for the safety subscale, 0.90 for the everydayness subscale and 0.88 for the community subscale, indicating a strong internal consistency reliability overall. Furthermore, the corrected item-total correlations for all items ranged from 0.54 to 0.79, indicating that each item correlated adequately with the total score and thus that the scale is homogenous without any item being redundant (Table 3).

Table 4 presents the results from the test-retest reliability assessment of the Chinese PCQ-S. The result of the Kp statistic for the overall scale scores was 0.85 ($P < 0.001$), indicating that the Chinese PCQ-S instrument has substantial reliability. For each subscale, the results varied from 0.75 to 0.82 ($P<0.001$). According to the Pearson's correlation coefficient analyses, the Chinese PCQ-S indicated high correlation between test and retest on all scale levels: on the sub-scales 'a climate of safety' ($r = 0.88$, $P<0.01$), 'a climate of everydayness' ($r=0.91$, $P<0.01$) and 'a climate of community' ($r=0.79$, $P<0.01$) as well as on the overall scale scores between test and

For peer review only - http://bmjopen.bmj.com/site/about/guidelines.xhtml

retest (r=0.93, P<0.01). A paired t-test also confirmed that there was no significant difference between the mean scores of the PCQ-S at the test and retest ratings (P>0.05). The ICC of the total score between the test and retest times was 0.97, providing further support that the scale had satisfactory test–retest reliability.

DISCUSSION

This is the first study to validate the PCQ-S in an Asian population, and the results of the present study indicated that the cross-culturally adapted Chinese version PCQ-S showed excellent reliability and validity for evaluating staff perceptions of person-centredness in Chinese hospital contexts, which enables further studies and international comparisons.

In this study, the English PCQ-S was cross-culturally adapted into the Chinese version and showed satisfactory psychometric properties (construct validity, test-retest reliability and internal consistency). During our translation of the English PCQ-S into Chinese, a minor cultural discrepancy was encountered and one item of the PCQ-S was therefore modified accordingly. In this instance, ‘peaceful’ was replaced by ‘harmonious’ as this word is closer to Chinese culture. Construct validity was estimated using PCA with varimax orthogonal rotation, resulting in a stable three-factor solution explaining 73.3% of the total variance. The ICC for the overall Chinese PCQ-S scale was 0.97 and for the three subscales was 0.93, 0.95 and 0.92, demonstrating that the test-retest reliability of the overall scale and different domains was excellent. Furthermore, strong internal consistency of the Chinese PCQ-S was demonstrated, as evidenced through a Cronbach’s alpha of 0.89 for the total scale, 0.87 for the safety subscale, 0.90 for the everydayness subscale and 0.88 for the community subscale.

This Chinese version of the PCQ-S showed the same sub-scale structure as the Swedish, Norwegian and Slovenian versions - a structure with the three subscales described above (a climate of safety, everydayness and community) consisting of 14

For peer review only - http://bmjopen.bmj.com/site/about/guidelines.xhtml

items. However, it showed a different structure with the English PCQ-S - a four-component structure (a climate of safety, everydayness, community and comprehensibility) consisting of 14 items, which may reflect a difference in cultural context. Even though the original English PCQ-S described a slightly different structure consisting of four subscales, the instrument developers have recently suggested keeping to the three subscale structure for scoring and comparison purposes as this confirms the theory on which the scale rests [20].

In the Chinese PCQ-S, the ICC (0.97) and Cronbach's alpha for the total scale (0.94) were much higher than in the Swedish (0.51 vs. 0.88) and English (0.75 vs. 0.89) versions, and the Cronbach's alpha for the total scale was also higher than it was in the Norwegian version (0.92). Due to the larger the sample size in our study, which differs from those in the above three studies, the Chinese PCQ-S may have stronger test-retest reliability and internal consistency compared to other language versions of the PCQ-S. The results demonstrated that the Chinese PCQ-S has good reproducibility and well maintains the properties of the original version, and can thus be used in Chinese hospital environments.

The following limitations of the present study should be noted. First, the study employed a convenience sampling method to select staff in palliative care from public hospitals, which may limit the ability to generalize the results to staff in general at Chinese hospitals or other staff working other health care contexts. Second, the Chinese PCQ-S questionnaire has been tested only in this hospital environment, and further psychometric testing of the scale in other settings like nursing homes would be helpful to enable further rigorous comparisons of Chinese PCQ-S in different contexts and settings. Third, the questionnaire had been translated only from the secondary English version, not from the original Swedish version. Fourth, with respect to the psychometric assessment of the PCQ-S, criterion-related validity, convergent validity and discriminative validity were not taken into account. Further study is needed to explore this in the future. Fifth, the back-translated version was not validated, due to

For peer review only - http://bmjopen.bmj.com/site/about/guidelines.xhtml

For peer review only

the cross-cultural adaptation.

Conclusion

The 14-item Chinese PCQ-S is a cross-culturally adapted version of the English PCQ-S, which showed excellent psychometric properties in terms of reliability and validity for evaluating staff perceptions of the person-centredness in Chinese hospital environments. Our results indicated that the Chinese version of the PCQ-S can be utilized for future measurement and development of person-centred care in China and for conducting cross-cultural international comparisons with for example Sweden.

Ethical approval and consent to participate

This study was approved by the Ethics Committee of Kunming Medical University. Oral and written informed consent was obtained from all persons participating in the study.

Availability of data and material

The datasets used and/or analysed during the current study are available from the corresponding author on request.

Competing interests

The authors declare that there are no conflicts of interest.

Consent for publication

Not applicable.

Funding

The study was supported by grants from the National Natural Science Fund of China (Grant number: 81611130077), the Swedish Research Council (Grant number: 2015-06243) and the Major Union Specific Project Foundation of Yunnan Provincial Science and Technology Department and Kunming Medical University (2016).

For peer review only - http://bmjopen.bmj.com/site/about/guidelines.xhtml

For peer review only

Authors' contributions

CL (Kunming Medical University, China) was responsible for the study design, data analysis, and drafting the paper. GA (Lund University, Sweden), TP (Kunming Medical University, China) and MK (the third people's hospital of Kunming, China) contributed to the study design and provided comments on the paper during the writing process. DE (La Trobe University, Australia) and LB (Lund University, Sweden) provided comments on the paper during the writing process. FH, ZJ and JY (the third people's hospital of Kunming, China) were responsible for the data collection.

Acknowledgements

We would like to thank Ms Huang Jingjing and Ms Wu Chao, two postgraduates of the School of Public Health, Kunming Medical University, who helped us with data collection and Magnus Persson, Lund University, Sweden for providing comments on the paper.

For peer review only

For peer review only - http://bmjopen.bmj.com/site/about/guidelines.xhtml

REFERENCES

1. Sixth National Population Census of the People's Republic of China. Beijing, China: National Bureau of Statistics of China 2011.
2. World population prospects: the 2012 revision. New York, United States: Department of Economic and Social Affairs (DoEaSA), Population Division, United Nations 2013.
3. Dong H-J, Wressle E, Marcusson J. Multimorbidity patterns of and use of health services by Swedish 85-year-olds: an exploratory study. *BMC Geriatr* 2013;13(1):120-20. doi: 10.1186/1471-2318-13-120
4. Liao C-C, Li C-R, Lee S-H, et al. Social support and mortality among the aged people with major diseases or ADL disabilities in Taiwan: A national study. *Arch Gerontol Geriatr* 2015;60(2):317-21. doi: 10.1016/j.archger.2014.11.007
5. Hsu CY, O'Connor M, Lee S. Understandings of death and dying for people of Chinese origin. *Death Stud* 2009;33(2):153-74. doi: 10.1080/07481180802440431
6. Chan CLW, Chow AYM. Death, dying and bereavement : a Hong Kong Chinese experience. Hong Kong: Hong Kong University Press 2006.
7. McGilton KS, Heath H, Chu CH, et al. Moving the agenda forward: a person-centred framework in long-term care. *Int J Older People Nurs* 2012;7(4):303-9. doi: 10.1111/opn.12010
8. Winefield H. MT, Clifford J., Farmer E. The search for reliable and valid measures of patient-centredness. *Psychol Health* 1996(11):811-24.
9. Edvardsson DW, B. Sandman, P O. Person-centred care of people with severe Alzheimer's disease: current status and ways forward. *Lancet Neurology* 2008(4):362. doi: 10.1016/S1474-4422(08)70063-2
10. McCormack B. Person-centredness in gerontological nursing: an overview of the literature. *J Clin Nurs* 2004;13(3a):31-8. doi: 10.1111/j.1365-2702.2004.00924.x
11. McCance T, Slater P, McCormack B. Using the caring dimensions inventory as an indicator of person-centred nursing. *J Clin Nurs* 2009;18(3):409-17. doi: 10.1111/j.1365-2702.2008.02466.x
12. Buckley C, McCormack B, Ryan A. Valuing narrative in the care of older people: A framework of narrative practice for older adult residential care settings. *J Clin Nurs* 2014;23(17-18):2565-77. doi: 10.1111/jocn.12472
13. Edvardsson D, Fetherstonhaugh D, Nay R. Promoting a continuation of self and normality: person-centred care as described by people with dementia, their family members and aged care staff. *J Clin Nurs* 2010;19(17-18):2611-18. doi: 10.1111/j.1365-2702.2009.03143.x
14. McCormack B, Dewing J, Breslin L, et al. Developing person-centred practice: nursing outcomes arising from changes to the care environment in residential settings for older people. *Int J Older People Nurs* 2010;5(2):93-107. doi: 10.1111/j.1748-3743.2010.00216.x
15. Crandall LG, White DL, Schuldheis S, et al. Initiating person-centered care practices in long-term care facilities. *J Gerontol Nurs* 2007;33(11):47-56.
16. McCormack B, McCance TV. Development of a framework for person-centred nursing. *J Adv Nurs* 2006;56(5):472-79. doi: 10.1111/j.1365-2648.2006.04042.x
17. Edvardsson D, Fetherstonhaugh D, McAuliffe L, et al. Job satisfaction amongst aged care staff: exploring the influence of person-centered care provision. *Int Psychogeriatr* 2011;23(8):1205-12. doi: 10.1017/S1041610211000159

For peer review only - http://bmjopen.bmj.com/site/about/guidelines.xhtml

18. McKeown J, Clarke A, Ingleton C, et al. The use of life story work with people with dementia to enhance person-centred care. *Int J Older People Nurs* 2010;5(2):148-58. doi: 10.1111/j.1748-3743.2010.00219.x
19. Bone C, Cheung G, Wade B. Evaluating person centred care and Dementia Care Mapping in a psychogeriatric hospital in New Zealand: a pilot study. *NZJOT* 2010;57(1):35-40.
20. Edvardsson D, Sandman PO, Rasmussen B. Construction and psychometric evaluation of the Swedish language Person-centred Climate Questionnaire - staff version. *J Nurs Manag* 2009;17(7):790-5. doi: 10.1111/j.1365-2834.2009.01005.x
21. Edvardsson D, Fetherstonhaugh D, Nay R, et al. Development and initial testing of the Person-centered Care Assessment Tool (P-CAT). *Int Psychogeriatr* 2010;22(1):101-8. doi: 10.1017/S1041610209990688
22. White DL, Newton-Curtis L, Lyons KS. Development and initial testing of a measure of person-directed care. *Gerontologist* 2008;48 Spec No 1:114-23.
23. Chappell N, Reid R, Gish J. Staff-based measures of individualized care for persons with dementia in long-term care facilities. *Dementia* 2007;6(4):527-47. doi: doi:10.1177/1471301207084372
24. Zimmerman S, Allen J, Cohen LW, et al. A measure of person-centered practices in assisted living: the PC-PAL. *J Am Med Dir Assoc* 2015;16(2):132-7. doi: 10.1016/j.jamda.2014.07.016
25. Edvardsson D, Sjogren K, Lindkvist M, et al. Person-centred climate questionnaire (PCQ-S): establishing reliability and cut-off scores in residential aged care. *J Nurs Manag* 2015;23(3):315-23. doi: 10.1111/jonm.12132
26. Bergland A, Kirkevold M, Edvardsson D. Psychometric properties of the Norwegian Person-centred Climate Questionnaire from a nursing home context. *Scand J Caring Sci* 2012;26(4):820-8. doi: 10.1111/j.1471-6712.2012.00979.x
27. Edvardsson D, Koch S, Nay R. Psychometric evaluation of the English language Person-centred Climate Questionnaire--staff version. *J Nurs Manag* 2010;18(1):54-60. doi: 10.1111/j.1365-2834.2009.01038.x
28. Vrbnjak D, Pahor D, Povalej Brzan P, et al. Psychometric testing of the Slovenian Person-centred Climate Questionnaire - staff version. *J Nurs Manag* 2017 doi: 10.1111/jonm.12479
29. Guillemin F. Cross-cultural adaptation and validation of health status measures. *Scand J Rheumatol* 1995;24(2):61-3.
30. Beaton DE, Bombardier C, Guillemin F, et al. Guidelines for the process of cross-cultural adaptation of self-report measures. *Spine (Phila Pa 1976)* 2000;25(24):3186-91.
31. Terwee CB, Bot SD, de Boer MR, et al. Quality criteria were proposed for measurement properties of health status questionnaires. *J Clin Epidemiol* 2007;60(1):34-42. doi: 10.1016/j.jclinepi.2006.03.012
32. Nunnally JC, Bernstein IH. Psychometric theory. New York: McGraw-Hill 1994.
33. Pett MA, Lackey NR, Sullivan JJ. Making sense of factor analysis : the use of factor analysis for instrument development in health care research. London: SAGE 2003.
34. Bland JM, Altman DG. Cronbach's alpha. *BMJ* 1997;314(7080):572.
35. Landis JR, Koch GG. The measurement of observer agreement for categorical data. *Biometrics* 1977;33(1):159-74.

For peer review only - http://bmjopen.bmj.com/site/about/guidelines.xhtml

Table 1 Demographic characteristics of the study sample (n=163)

Characteristics	n (%)
Gender	
Female	151 (92.6)
Male	12 (7.4)
Age (years)	
18-30	95 (58.3)
31-39	33 (20.2)
⩾40	35 (21.5)
Level of education	
High school	7 (4.3)
Secondary school	37 (22.7)
Junior college	65 (39.9)
Bachelor or higher	54 (33.2)
Ethnicity	
Han	118 (72.4)
Minorities	45 (27.6)
Healthcare staff	
Registered nurse	101 (62.0)
Enrolled nurse	29 (17.8)
Physician	33 (20.2)

For peer review only

Table 2 Rotated component matrix for PCA of the Chinese PCQ-S (n=163)

Item number	Item content	Factor loadings		
		Subscale 1: A climate of safety	Subscale 2: A climate of everydayness	Subscale 3: A climate of community
1	A place where I feel welcome	0.83		
2	A place where I feel acknowledged as a person	0.84		
3	A place where I feel I can be myself	0.58		
4	A place where the patients are in safe hands	0.66		
5	A place where the staff use a language that the patients can understand	0.60		
6	A place which feels homely even though it is in an institution		0.82	
7	A place where there is something nice to look at		0.81	
8	A place where it is quiet and peaceful		0.78	
9	A place where it is possible to get unpleasant thoughts out of your head		0.74	
10	A place which is neat and clean		0.68	
11	A place where it is easy for the patients to keep in contact with their loved ones			0.64
12	A place where it is easy for the patients to receive visitors			0.87
13	A place where it is easy for the patients to talk to the staff			0.85
14	A place where the patients have someone to talk to if they so wish			0.66
Total variance explained (%)	73.3 (total 3 subscales)	55.6	9.5	8.2
Cronbach's alpha	0.94 (total 14 items)	0.87	0.90	0.88

For peer review only - http://bmjopen.bmj.com/site/about/guidelines.xhtml

Table 3 Item performance and reliability test of the Chinese PCQ-S (n=163)

Item number	Item content	Mean±SD	Corrected item: total correction	Cronbach's alpha if item deleted
1	A place where I feel welcome	4.04±0.93	0.62	0.93
2	A place where I feel acknowledged as a person	4.07±0.92	0.54	0.93
3	A place where I feel I can be myself	3.58±1.32	0.70	0.93
4	A place where the patients are in safe hands	4.06±0.96	0.72	0.93
5	A place where the staff use a language that the patients can understand	3.90±1.01	0.72	0.93
6	A place which feels homely even though it is in an institution	3.80±1.13	0.77	0.93
7	A place where there is something nice to look at	3.60±1.15	0.76	0.93
8	A place where it is quiet and peaceful	3.80±1.04	0.78	0.93
9	A place where it is possible to get unpleasant thoughts out of your head	3.20±1.34	0.66	0.93
10	A place which is neat and clean	3.85±1.01	0.70	0.93
11	A place where it is easy for the patients to keep in contact with their loved ones	3.88±1.03	0.79	0.93
12	A place where it is easy for the patients to receive visitors	3.40±1.36	0.59	0.93
13	A place where it is easy for the patients to talk to the staff	3.72±1.16	0.71	0.93
14	A place where the patients have someone to talk to if they so wish	3.94±1.03	0.67	0.93

For peer review only - http://bmjopen.bmj.com/site/about/guidelines.xhtml

Table 4 Test-retest reliability of the Chinese PCQ-S (n=163)

Scale dimension	1st test (Mean±SD)	2nd test (Mean±SD)	P	Weighted kappa (Kp)	Pearson correlation coefficient (r)	ICC (95% CI)
A climate of safety	19.7±4.2	19.8±4.0	0.30	0.77	0.88	0.93 (0.91, 0.95)
A climate of everydayness	18.3±4.8	18.1±4.9	0.38	0.82	0.91	0.95 (0.93, 0.96)
A climate of community	15.0±4.0	14.7±4.1	0.18	0.75	0.79	0.92 (0.89, 0.94)
Overall scale	52.9±11.4	52.6±11.7	0.40	0.85	0.93	0.97 (0.95-0.98)

For peer review only - http://bmjopen.bmj.com/site/about/guidelines.xhtml

STROBE Statement—Checklist of items that should be included in reports of ***cross-sectional studies***

	Item No.	Recommendation	Page No.	Relevant text from manuscript
**Title and abstract**	1	(*a*) Indicate the study's design with a commonly used term in the title or the abstract	0	cross-sectional design
		(*b*) Provide in the abstract an informative and balanced summary of what was done and what was found	1-2	Results, Conclusions
**Introduction**				
Background/rationale	2	Explain the scientific background and rationale for the investigation being reported	3-5	Introduction
Objectives	3	State specific objectives, including any prespecified hypotheses	5	The purpose of this study
**Methods**				
Study design	4	Present key elements of study design early in the paper	5	Methods
Setting	5	Describe the setting, locations, and relevant dates, including periods of recruitment, exposure, follow-up, and data collection	-	Sample and Participants, Data collection
Participants	6	(*a*) *Cohort study*—Give the eligibility criteria, and the sources and methods of selection of participants. Describe methods of follow-up<br>*Case-control study*—Give the eligibility criteria, and the sources and methods of case ascertainment and control selection. Give the rationale for the choice of cases and controls<br>*Cross-sectional study*—Give the eligibility criteria, and the sources and methods of selection of participants	6-7	Sample and Participants, inclusion criteria
		(*b*) *Cohort study*—For matched studies, give matching criteria and number of exposed and unexposed<br>*Case-control study*—For matched studies, give matching criteria and the number of controls per case		
Variables	7	Clearly define all outcomes, exposures, predictors, potential confounders, and effect modifiers. Give diagnostic criteria, if applicable	5-8	Results
Data sources/ measurement	8*	For each variable of interest, give sources of data and details of methods of assessment (measurement). Describe comparability of assessment methods if there is more than one group	5-6	Psychometric evaluation
Bias	9	Describe any efforts to address potential sources of bias	11	limitation
Study size	10	Explain how the study size was arrived at	6	Sample size

Continued on next page

For peer review only - http://bmjopen.bmj.com/site/about/guidelines.xhtml

Quantitative variables	11	Explain how quantitative variables were handled in the analyses. If applicable, describe which groupings were chosen and why	7-8	Psychometric evaluation
Statistical methods	12	(*a*) Describe all statistical methods, including those used to control for confounding	7-8	Psychometric evaluation
		(*b*) Describe any methods used to examine subgroups and interactions		
		(*c*) Explain how missing data were addressed		
		(*d*) *Cohort study*—If applicable, explain how loss to follow-up was addressed<br>*Case-control study*—If applicable, explain how matching of cases and controls was addressed<br>*Cross-sectional study*—If applicable, describe analytical methods taking account of sampling strategy		
		(*e*) Describe any sensitivity analyses		
**Results**				
Participants	13*	(a) Report numbers of individuals at each stage of study—eg numbers potentially eligible, examined for eligibility, confirmed eligible, included in the study, completing follow-up, and analysed	6	All staff (n=182) on duty<br>163 consented to participate
		(b) Give reasons for non-participation at each stage		
		(c) Consider use of a flow diagram		
Descriptive data	14*	(a) Give characteristics of study participants (eg demographic, clinical, social) and information on exposures and potential confounders	8	Demographic characteristics of the study group
		(b) Indicate number of participants with missing data for each variable of interest		
		(c) *Cohort study*—Summarise follow-up time (eg, average and total amount)		
Outcome data	15*	*Cohort study*—Report numbers of outcome events or summary measures over time		
		Case-control study—Report numbers in each exposure category, or summary measures of exposure		
		Cross-sectional study—Report numbers of outcome events or summary measures	8-9	Results
Main results	16	(*a*) Give unadjusted estimates and, if applicable, confounder-adjusted estimates and their precision (eg, 95% confidence interval). Make clear which confounders were adjusted for and why they were included	8-9	
		(*b*) Report category boundaries when continuous variables were categorized	-	
		(*c*) If relevant, consider translating estimates of relative risk into absolute risk for a meaningful time period	-	

Continued on next page

For peer review only - http://bmjopen.bmj.com/site/about/guidelines.xhtml

Other analyses	17	Report other analyses done—eg analyses of subgroups and interactions, and sensitivity analyses	-	
**Discussion**				
Key results	18	Summarise key results with reference to study objectives	10	Discussion
Limitations	19	Discuss limitations of the study, taking into account sources of potential bias or imprecision. Discuss both direction and magnitude of any potential bias	11	The following limitations of the present study should be noted
Interpretation	20	Give a cautious overall interpretation of results considering objectives, limitations, multiplicity of analyses, results from similar studies, and other relevant evidence	10-11	
Generalisability	21	Discuss the generalisability (external validity) of the study results	11	First, the study employed a convenience sampling method to select staff in palliative care from public hospitals, which may limit the ability to generalize the results to a larger population of staff in Chinese hospitals and beyond.
**Other information**				
Funding	22	Give the source of funding and the role of the funders for the present study and, if applicable, for the original study on which the present article is based	12	Funding

-

*Give information separately for cases and controls in case-control studies and, if applicable, for exposed and unexposed groups in cohort and cross-sectional studies.

Note: An Explanation and Elaboration article discusses each checklist item and gives methodological background and published examples of transparent reporting. The STROBE checklist is best used in conjunction with this article (freely available on the Web sites of PLoS Medicine at http://www.plosmedicine.org/, Annals of Internal Medicine at http://www.annals.org/, and Epidemiology at http://www.epidem.com/). Information on the STROBE Initiative is available at www.strobe-statement.org.

For peer review only

For peer review only - http://bmjopen.bmj.com/site/about/guidelines.xhtml
